# Efficient experimental design for uncertainty reduction in gene regulatory networks

**DOI:** 10.1186/1471-2105-16-S13-S2

**Published:** 2015-09-25

**Authors:** Roozbeh Dehghannasiri, Byung-Jun Yoon, Edward R Dougherty

**Affiliations:** 1 Department of Electrical and Computer Engineering, Texas A&M University, College Station, TX 77843, USA; 2 Center for Bioinformatics and Genomic Systems Engineering, Texas A&M University, College Station, TX 77845, USA; 3 College of Science and Engineering, Hamad bin Khalifa University (HBKU), Doha, Qatar

**Keywords:** Experimental design, gene regulatory networks, mean objective cost of uncertainty, objective-based network reduction, Boolean networks, structural intervention

## Abstract

**Background:**

An accurate understanding of interactions among genes plays a major role in developing therapeutic intervention methods. Gene regulatory networks often contain a significant amount of uncertainty. The process of prioritizing biological experiments to reduce the uncertainty of gene regulatory networks is called experimental design. Under such a strategy, the experiments with high priority are suggested to be conducted first.

**Results:**

The authors have already proposed an optimal experimental design method based upon the objective for modeling gene regulatory networks, such as deriving therapeutic interventions. The experimental design method utilizes the concept of mean objective cost of uncertainty (MOCU). MOCU quantifies the expected increase of cost resulting from uncertainty. The optimal experiment to be conducted first is the one which leads to the minimum expected remaining MOCU subsequent to the experiment. In the process, one must find the optimal intervention for every gene regulatory network compatible with the prior knowledge, which can be prohibitively expensive when the size of the network is large. In this paper, we propose a computationally efficient experimental design method. This method incorporates a network reduction scheme by introducing a novel cost function that takes into account the disruption in the ranking of potential experiments. We then estimate the approximate expected remaining MOCU at a lower computational cost using the reduced networks.

**Conclusions:**

Simulation results based on synthetic and real gene regulatory networks show that the proposed approximate method has close performance to that of the optimal method but at lower computational cost. The proposed approximate method also outperforms the random selection policy significantly. A MATLAB software implementing the proposed experimental design method is available at http://gsp.tamu.edu/Publications/supplementary/roozbeh15a/.

## Background

A key area in the field of translational genomics is to derive therapeutic intervention that can beneficially alter cell dynamics in such a way as to avoid cancerous phenotypes. The first step to derive therapeutic interventions is to understand the regulatory relationships among genes. The interactions among genes are studied in the context of gene regulatory networks (GRNs). GRN models often possess high uncertainty. This inherent uncertainty might be due to many factors such as the complex nature of biological phenomena, lack of enough training data, etc. Uncertainty in GRNs can affect the accuracy and performance of the therapeutic interventions. Therefore, biologists aim at reducing uncertainty of GRN model via conducting additional biological experiments. However, there are many limitations for carrying out experiments. Biological experiments are usually expensive and time consuming as they need to be done on living organisms. On the other hand, the resources are limited which prevents researchers from conducting all experiments they need for identification of GRN. Thus, it is prudent to prioritize potential experiments based on the information they provide and then conduct the most informative. This process is called *experimental design*.

Boolean networks (BNs) have been extensively used for the study of GRNs. They have been shown to be effective in capturing the multivariate relationships among entities within a cell. BNs also facilitate the use of the well-studied Markov decision theory for deriving beneficial interventions. To date, intervention methods for BNs have been categorized in two different groups: structural interventions, and dynamical interventions. While structural interventions [1-4] permanently change the behavior of a network via one-time change in the underlying regulatory structure, the goal in dynamical interventions [5-8] is to interfere with the signaling of GRN through flipping or not flipping the expression state of genes over time. The assumption behind most of these intervention methods is that the network being studied is perfectly known. Thus, presence of uncertainty can degrade the performance of the intervention methods.

From a translational perspective it is crucial to reduce uncertainty pertinent to the objective, such as intervention. Hence, uncertainty should be quantified in such a way that the objective for modeling GRN is taken into account. Mean objective cost of uncertainty (MOCU) [9] quantifies the uncertainty of model in terms of the expected increased cost due to the presence of uncertainty. An experimental design method based on MOCU has been proposed in [10]. The experimental design method in [10] evaluates the effect of each potential experiment in reducing the model uncertainty, which is measured in terms of MOCU, and suggests that the experiment which results in the minimum expected remaining MOCU should be conducted first. The long-run performance of the experimental design method in [10] is guaranteed to be optimal in terms of reducing the error of interventions obtained after conducting the chosen experiments.

Although, the method in [10] is optimal, it is computationally expensive. Because our final objective is to improve the performance of the therapeutic interventions, method in [10] involves finding optimal interventions for all networks which are compatible with the prior knowledge. Finding optimal interventions is computationally expensive whose complexity grows exponentially with the number of genes in network. Therefore, the computational complexity of finding optimal experiment can be prohibitively high for large networks. Thus, it is inevitable to construct a smaller network via deleting some genes from the original large size network and then estimate the optimal interventions using the resulting reduced network. Generally the goal in network reduction methods is to produce networks of smaller size while the dynamical behavior of the original network is preserved. There have been some efforts for network reduction to reduce the complexity of designing interventions [11-13].

In this paper, we propose a novel cost function for the gene deletion process which takes into account the disruption in the order of potential experiments when they are ranked according to the experimental design method in [10]. Since experiments are ranked based upon the expected remaining MOCU or the MOCU that is expected to remain after performing the experiment, we desire that the network reduction step has a low effect on the expected remaining MOCU corresponding to the potential experiments. When the gene (or genes) suggested by the cost function are deleted from network, the optimal (and robust) interventions are estimated using the reduced networks and then they are used for calculating expected remaining MOCU for prioritizing potential experiments. We show the effectiveness of our proposed cost effective experimental design method through simulations on synthetic and real networks. The simulation results verify that our method can perform comparable to the optimal experimental design method in [10] with much lower computations.

MOCU-based optimal experimental design is very general and does not even require a Markovian network [10]. As we will see, finding the best gene to delete is also very general; however, once the genes are deleted, the regulatory structure of the original network must be mapped onto a corresponding regulatory structure on the reduced network, an optimal intervention must be found on the reduced network, and that intervention must be induced to the full network. Reduction and inducement are nontrivial and depend on the nature of the regulatory structure. The problem has been addressed for Boolean networks in [13], to which we refer, and a theoretical analysis is given in [11], where it is noted that the methodology applies to probabilistic Boolean networks (PBNs) [14] by applying the reduction to each of constituent BN of the PBN. Moreover, whereas we will restrict intervention to rank-one perturbations [2], which provide a one-time alteration of the regulatory logic, the reduction-inducement paradigm applies to other forms of intervention [11,13].

## Methods

### Boolean networks

Gene regulatory network models are increasingly used as a tool to study interactions among genes [15,16]. Boolean networks (BNs) [17] and probabilistic Boolean networks (PBNs) [14] are widely used models for GRNs that have been shown to be effective in capturing these interactions [18-24]. A Boolean network on *n *genes is defined by a pair (**V**, **F**). **V **= {*X*_1_, *X*_2_,..., *X_n_*} is a set of binary variables that represent the expression state of genes with *X_i _*= 0 and *X_i _*= 1 corresponding to gene *i *being OFF and ON, respectively. The ON state means that a gene is translated into a protein whereas the OFF state means that the gene is not translated. It has been shown that significant biological information can be extracted from binarized gene expression data [25,26]. The gene values at each time step are updated according to the list of Boolean functions **F **= {*f*_1_, *f*_2_,..., *f_n_*} with *X_i _*= *f_i_*(*X*_*i*1_, *X*_*i*2_,..., $X_{ik_{i}}$) where *X*_*i*1_, *X*_*i*2_,..., $X_{ik_{i}}$ are *k*_*i *_predictor genes for *X_i_*. The state of the BN at time *t *is the vector *X*(*t*) = (*X*_1_(*t*), *X*_2_(*t*),..., *X_n_*(*t*)), which is called *gene activity profile *(GAP). A BN with *n *genes possesses 2*^n ^*different states. The state of a BN for the next time instant *t *+ 1 is updated as *X*(*t *+ 1) = **F**(*X*(*t*)). In a Boolean network with perturbation (BNp), there is a perturbation probability of each gene flipping its value independently from other genes. Thus, in the presence of perturbation *X*(*t*+1) = **F**(*X*(*t*)) with probability (1 − *p*)*^n ^*and with probability 1 − (1 − *p*)*^n ^*at least one gene flips its value. The sequence of states of a BNp over time can be viewed as the states of a Markov chain with transition probability matrix (TPM) $\text{P = }{\left[p_{ij}\right]}_{i,j=1}^{2^{n}}$ where *p_ij _*is the probability that state *i *transitions into state *j*. Positive perturbation probability *p >*0 makes the Markov chain ergodic and irreducible, thereby possessing a unique steady-state distribution (SSD) **π**^*T *^= **π**^*T *^**P**, where *π_i _*is the steady-state probability of state *i *and *T *is the transpose operator. More details on how to compute TPM can be found in [2]. The long-run behavior of a BNp is characterized by its SSD, which is indicative of phenotypes. From a translational perspective, the states of a BNp can be partitioned into the set of desirable states *D *containing those states associated with healthy phenotypes and undesirable states *U *containing states associated with cancerous phenotypes. The goal of therapeutic interventions is to drive the network away from undesirable states and consequently reduce the steady-state probability mass of undesirable states, *π_U _*= ∑*_U_π*_i_. Without loss of generality regarding the experimental design analysis, in this paper we will assume that the undesirable states are determined by a single gene, called the "target gene" with the aim of intervention being to flip an undesirable value of the target gene to a desirable value. Two intervention approaches are commonly considered: (1) Structural interventions change the long-run behavior of the network by altering its underlying rule-based structure [1-4], and (2) dynamical interventions affect the dynamical evolution of the network by manipulating the expression states of some "control genes" [5-8].

This paper considers optimal structural intervention via rank-one function perturbations [2]. In a rankone function perturbation intervention, the relation between the TPM before intervention, **P**, and after intervention, $\tilde{P}$, is $\tilde{P}=P+ab^{T}$ where **a **and **b **are two arbitrary vectors satisfying **b***^T ^***e **= 0, where **e **is an all unity column vector. Single-gene perturbation, a special case of rank-one function perturbation, changes the regulatory function **F **for only one state. If originally **F**(*u*) = *w*, then the single-gene perturbation (*u, v*) means that for the regulatory function $\tilde{F}$after intervention, $\tilde{F}\left(u\right)=v$ and $\tilde{F}\left(i\right)=F\left(i\right)$ for *i ≠ u*. For this intervention, all entries of **P **and $\tilde{P}$ would be identical except for two entries. ${\tilde{p}}_{uw}=p_{uw}-{\left(1-p\right)}^{n}$ and ${\tilde{p}}_{uv}=p_{uv}+{\left(1-p\right)}^{n}$. The steady-state probability of state *i *after intervention (*u, v*) is found using the following equation:

(1) $${\tilde{\pi }}_{i}\left(u,v\right)=\pi_{i}+\frac{{\left(1-p\right)}^{n}\pi_{u}\left(z_{vi}-z_{wi}\right)}{1-{\left(1-p\right)}^{n}\left(z_{vu}-z_{wu}\right)},$$

where ${\tilde{\pi }}_{i}\left(u,v\right)$ is the steady-state probability mass of state *i *after single-gene perturbation (*u, v*) and *z_vi_*, *z_wi_*, *z_vu_*, and *z_wu _*belong to the fundamental matrix **Z **= [**I***−***P**+**e*****π**^T^*]^−1 ^where **I **is the *n *× *n *identity matrix. The undesirable steady state probability mass after single-gene function perturbation (*u, v*) is ${\tilde{\pi }}_{U}\left(u,v\right)=\sum_{i\in U}{\tilde{\pi }}_{i}\left(u,v\right)$. To find the optimal intervention, one needs to search among all possible 2*^n ^*× 2*^n ^*state pairs (*u, v*).

### Optimal experimental design

This section briefly reviews the optimal experimental design method in [10]. Suppose *θ*_1_, *θ*_2_,..., *θ_k _*are *k *uncertain parameters in the network model. They compose an *uncertainty vector θ *= (*θ*_1_, *θ*_2_,..., *θ_k _*), the set of all such vectors being denoted Θ. The collection of all networks corresponding to *θ *∈ Θ constitute an uncertainty class, denoted by Θ. Let Ψ be a class of potential interventions (in this paper, Ψ consisting of all single-gene function perturbations). Let *ξ_θ _*(*ψ*) denote the error of applying intervention *ψ *∈ Ψ to the network with uncertainty vector *θ *∈ Θ. In case of intervention, *ξ_θ _*(*ψ*) can (and will) be the steady-state probability mass of undesirable states after intervention, denoted ${\tilde{\pi }}_{U,\theta }\left(\psi \right)$. For a network with uncertainty vector *θ*, the optimal intervention *ψ*(*θ*) is defined by

(2) $$\psi \left(\theta \right)=\underset{\psi \in \Psi }{\text{arg}\;\text{min}}\;\xi_{\theta }\left(\psi \right).$$

In the presence of uncertainty, we desire an intervention that performs optimally on average across the uncertainty class. The intrinsic Bayesian robust (IBR) intervention $\psi_{IBR}\left(\Theta \right)$ is defined by

(3) $$\psi_{IBR}\left(\Theta \right)=\underset{\psi \in \Psi }{\text{arg}\;\text{min}}\;E_{\theta }\left[\xi_{\theta }\left(\psi \right)\right],$$

where *E_θ _*[•] denotes an expectation relative to the probability density function *f *(*θ*) governing the uncertainty class Θ. Note that although $\psi_{IBR}\left(\Theta \right)$ has the best expected performance across the uncertainty class, it is not guaranteed to have the best performance for each network inside the uncertainty class. The *mean objective cost of uncertainty *(MOCU) relative to Θ and Ψ is defined by [9]

(4) $$M_{\Psi }(\Theta )=E_{\theta }\left[\xi_{\theta }(\psi_{IBR}(\Theta ))-\xi_{\theta }(\theta ))\right],$$

the expression inside the expectation being called the *objective cost of uncertainty*. MOCU is the expected difference between the performance of the robust intervention and the optimal intervention for each network inside Θ. MOCU is an uncertainty quantification scheme which measures uncertainty by taking into account the objective of operator design, herein, network intervention. MOCU can effectively capture the amount of uncertainty and tries to quantify the uncertainty based on a specific objective (such as intervention) to gauge how much it is going to affect the objective [9].

MOCU has been used to design experiments that can maximally enhance the performance of the interventions [10]. Suppose that there are *k *potential experiments *E*_1_, *E*_2_,..., *E_k _*corresponding to *k *uncertain parameters *θ*_1_, *θ*_2_,..., *θ_k _*. It is assumed that experiment *E_i_*, which might be a complex experiment consisting of several sub-experiments, fully identifies *θ_i_*. The goal of experimental design is to find out which experiment *E_i_*, 1 ≤ *i *≤ *k *should be conducted first, or how to rank potential experiments effectively. Although we do not know the actual outcome of an experiment before conducting it, we do know the possible outcomes. Suppose the outcome of experiment *E_i _*is *ϕ*. We define the remaining MOCU, given *θ_i _*= *ϕ*, as

$$M_{\psi }\left(\Theta_{i,\phi }\right)=E_{\theta_{\phi }^{\left(i\right)}}\left[\xi_{\theta_{\phi }^{\left(i\right)}}\left(\psi_{IBR}\left(\Theta_{i,\phi }\right)\right)-\xi_{\theta_{\phi }^{\left(i\right)}}\left(\psi \left(\theta_{\phi }^{\left(i\right)}\right)\right)\right],$$

where $\theta_{\phi }^{\left(i\right)}$, called the *conditional uncertainty vector*, has *i*th parameter equal to  ϕ and other parameters are still random, and Θ*_i,ϕ _*is the *remaining uncertainty class *when *θ_i _*= *ϕ*, i.e., Θ*_i,ϕ _*= {*θ|θ *∈ Θ, *θ_i_* = *ϕ*}. The expectation is taken over the conditional density function $f\left(\theta_{\phi }^{\left(i\right)}\right)=f\left(\theta |\theta_{i}=\phi \right)$ that governs the remaining uncertainty class Θ*_i,ϕ_*. $\psi_{IBR}\left(\Theta_{i,\phi }\right)$ is the robust intervention for Θ*_i,ϕ _*and is found in a similar way as (3).

Corresponding to each experiment *E_i_*, we define the expected remaining MOCU after conducting that experiment as:

(6) $$\begin{array}{c} M_{\Psi }\left(\Theta ,i\right)\\ \;=E_{\theta_{i}}\left[M_{\Psi }\left({\Theta_{i,\theta }}_{{}_{i}}\right)\right]\\ \;=E_{\theta_{i}}\left[E_{\theta_{\theta_{i}}^{\left(i\right)}}\left[\xi_{\theta_{\theta_{i}}^{\left(i\right)}}\left(\psi_{IBR}\left({\Theta_{i,\theta }}_{{}_{i}}\right)\right)-\xi_{\theta_{\theta_{i}}^{\left(i\right)}}\left(\psi \left(\theta_{\theta_{i}}^{\left(i\right)}\right)\right)\right]\right], \end{array}$$

where *E_θi_*[•] is the expectation relative to the marginal density function *f*(*θ_i_*) of uncertain parameter *θ_i_*. Then we find the minimum expected remaining MOCU:

$$i^{*}=\underset{i=1,2,...,k}{\text{arg}\;\text{min}\;}M_{\Psi }\left(\Theta ,i\right).$$

E_i*_ is the optimal experiment to be conducted first [10].

### Approximate experimental design method

According to (5) and (6), calculating the expected remaining MOCU requires finding the optimal intervention *ψ*(*θ*) for each *θ *∈ Θ and the robust intervention $\psi_{IBR}\left(\Theta_{i,\phi }\right)$ for each possible remaining uncertainty class. The complexity of finding optimal interventions grows exponentially with network size *n*. For finding an optimal single-gene structural intervention, we need to search among all possible 2*^n ^*× 2*^n ^*state pairs and calculate the new steady-state probability ${\tilde{\pi }}_{i}$ for each state *i *in the set of undesirable states *U*. Thus, the complexity is  O(2^3*n*^). This heavy computational cost motivates us to reduce the size of network in order to reduce the complexity of finding optimal interventions, thereby reducing the complexity of the experimental design.

Assuming that gene *g *is deleted from a network with regulatory function **F**, we define a regulatory function **F***_red _*for the reduced network. Doing this for each network with uncertainty vector *θ *in Θ produces the uncertainty class, Θ^g^, of reduced networks via the mapping *θ → θ^g^*.

To approximate the optimal intervention for a network in Θ, we use the corresponding network in Θ*^g^*, find the optimal intervention for the reduced network *ψ*(*θ^g^*), and then induce the intervention to the original network in Θ. This approximate optimal intervention denoted by *ψ*(*θ*;*g*) is called the *induced optimal intervention*. Also, to find the induced robust intervention, $\psi_{IBR}^{ind}\left(\Theta ;g\right)$, for Θ, first we find the robust intervention, $\psi_{IBR}\left(\Theta^{g}\right)$, for Θ*^g ^*using (3) and then find the induced robust intervention $\psi_{IBR}^{ind}\left(\Theta ;g\right)$ from $\psi_{IBR}\left(\Theta^{g}\right)$.

**Figure 1:**
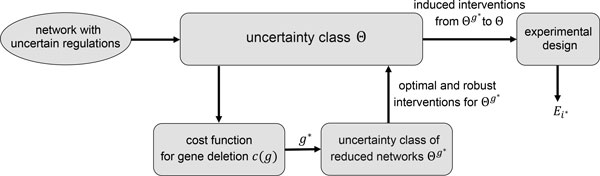
**An illustrative view of the general approach of the proposed approximate experimental design method**.

As illustrated in Figure 1, in the proposed approximate experimental design method, we find the best gene *g^∗ ^*for deletion via a novel cost function *c*(*g*) and then obtain the induced optimal and robust interventions needed for the MOCU calculations in the experimental design step by inducing interventions from uncertainty class of reduced networks Θ*^g^∗^^* to the original uncertainty class Θ.

We now aim to find a gene whose deletion results in minimum degradation in the experimental design process. Keeping in mind that the experimental design is based on the expected remaining MOCU for potential experiments, let $M_{\Psi }^{g}\left(\Theta_{i,\theta_{i}}\right)$ be the remaining MOCU when uncertain parameter *i *has value *θ_i _*and we delete gene *g*,

(8) $$M_{\Psi }^{g}\left(\Theta_{i,\theta_{i}}\right)=E_{\theta_{\theta_{i}}^{\left(i\right)}}\left[\xi_{\theta_{\theta_{i}}^{\left(i\right)}}\left(\psi_{IBR}^{ind}\left({\Theta_{i,\theta }}_{{}_{i};}g\right)\right)-\xi_{\theta_{\theta_{i}}^{\left(i\right)}}\left(\psi \left(\theta_{\theta_{i}}^{\left(i\right)}\right)\right)\right].$$

We define the cost of deleting gene *g *by

(9) $$c\left(g\right)=\overset{k}{\underset{i=1}{\sum }}|M_{\Psi }^{g}\left(\Theta ,i\right)-M_{\Psi }\left(\Theta ,i\right)|,$$

where

(10) $$M_{\Psi }^{g}\left(\Theta ,i\right)=E_{\theta_{i}}\left[M_{\Psi }^{g}\left({\Theta_{i,\theta }}_{{}_{i}}\right)\right].$$

The gene *g^∗ ^*minimizing the cost function in (9) is selected for deletion:

(11) $$g^{*}=\underset{g\in 1,2,...,n}{\text{arg}\;\text{min}}\;c\left(g\right).$$

The intuition behind this cost function is that our choice of optimal experiment is based upon the expected remaining MOCU corresponding to each experiment. Therefore, we desire that the network reduction step has minimum effect on these quantities. Deleting genes increases the inherent uncertainty of the network because the induced robust intervention cannot perform better than the original robust intervention on average. We want to reduce this increase in the uncertainty of model caused by network reduction. Since

(12) $$E_{\theta_{\theta_{i}}^{\left(i\right)}}\left[\xi_{\theta_{\theta_{i}}^{\left(i\right)}}\left(\psi_{IBR}^{ind}\left({\Theta_{i,\theta }}_{{}_{i};}g\right)\right)\right]\geq E_{\theta_{\theta_{i}}^{\left(i\right)}}\left[\xi_{\theta_{\theta_{i}}^{\left(i\right)}}\left(\psi_{IBR}\left({\Theta_{i,\theta }}_{{}_{i}}\right)\right)\right],$$

$M_{\Psi }^{g}\left(\Theta ,i\right)\geq M_{\Psi }\left(\Theta ,i\right).$ Hence, we can omit the absolute value operator in (9) to obtain

(13) $$\begin{array}{c} g^{*}=\underset{g}{\text{arg}\;\text{min}}\overset{k}{\underset{i=1}{\sum }}\left(M_{\Psi }^{g}\left(\Theta ,i\right)-M_{\Psi }\left(\Theta ,i\right)\right)\\ =\underset{g}{\text{arg}\;\text{min}}\overset{k}{\underset{i=1}{\sum }}M_{\Psi }^{g}\left(\Theta ,i\right), \end{array}$$

where the second equality follows from the fact that *M*_Ψ_(*θ*,*i*) does not depend on the gene being deleted. Expanding $M_{\Psi }^{g}\left(\Theta ,i\right)$ yields

(14) $$\begin{array}{c} g^{*}=\underset{g}{\text{arg}\;\text{min}}\left\{\overset{k}{\underset{i=1}{\sum }}E_{\theta_{i}}\left[E_{\theta_{\theta_{i}}^{\left(i\right)}}\left[\xi_{\theta_{\theta_{i}}^{\left(i\right)}}\left(\psi_{IBR}^{ind}\left({\Theta_{i,\theta }}_{{}_{i}};g\right)\right)-\xi_{\theta_{\theta_{i}}^{\left(i\right)}}\left(\psi \left(\theta_{\theta_{i}}^{\left(i\right)}\right)\right)\right]\right]\right\}\\ \;=\underset{g}{\text{arg}\;\text{min}}\overset{k}{\underset{i=1}{\sum }}E_{\theta_{i}}\left[E_{\theta_{\theta_{i}}^{\left(i\right)}}\left[\xi_{\theta_{\theta_{i}}^{\left(i\right)}}\left(\psi_{IBR}^{ind}\left({\Theta_{i,\theta }}_{{}_{i}};g\right)\right)\right]\right]. \end{array}$$

The minimization problem in (14) is equivalent to the one in (11). Based on the cost function in (14), for each gene *g*, we find the expected performance of the induced robust intervention $\psi_{IBR}^{ind}\left({\Theta_{i,\theta }}_{{}_{i}};g\right)$ across the remaining uncertainty class Θ*_i,θi_*, then take the expectation of this average performance relative to the marginal distribution of the uncertain parameter *θ_i_*, and finally sum all values found for the *k *uncertain parameters.

**Algorithm 1 **Approximate experimental design

1: **input**: Θ, Ψ, *f *(*θ*), *θ *= (*θ*_1_,..., *θ_k_*)

2: **output**: E_i*_, i^∗ ^∈ {1,..., *k*}: the estimated optimal experiment to be conducted first

3: **for ***g *= 1 : *n ***do**

4:   *cost*(*g*) ← 0

5:   **for ***i *= 1 : *k ***do**

6:      **for all ***θ_i _***do**

7:         build remaining uncertainty class of reduced networks $\Theta_{i,\theta_{i}}^{g}$

8:         compute conditional density function $f\left(\theta_{\theta_{i}}^{\left(i\right)}\right)$

9:         find induced robust intervention $\Psi_{IBR}^{ind}\left(\Theta_{i,\theta_{i}};g\right)$

10:         $h_{g}\left(\theta_{i}\right)\leftarrow E_{\theta_{\theta_{i}}^{\left(i\right)}}\left[\xi_{\theta_{\theta_{i}}^{\left(i\right)}}\left(\psi_{IBR}^{ind}\left({\Theta_{i,\theta }}_{{}_{i}};g\right)\right)\right]$

11:      *cost*(*g*) *← cost*(*g*) + $E_{\theta_{i}}$[*h_g _*(*θ_i_*)]

12: $g^{*}\leftarrow \underset{g=1,2,...,n}{\text{arg}\;\text{min}}\;\mathrm{cost}\left(g\right)$

13: **for ***i *= 1 : *k ***do**

14:   **for all ***θ_i _***do**

15:      build remaining uncertainty class $\Theta_{i,\theta_{i}}$

16:      compute conditional density function $f\left(\theta_{\theta_{i}}^{\left(i\right)}\right)$

17:      compute $M_{\Psi }^{g*}\left(\Theta_{i,\theta_{i}}\right)$ via equation (5) using $\Psi_{IBR}^{ind}\left(\Theta_{i,\theta_{i}};g^{*}\right)$ and $\Psi^{ind}\left(\theta_{\theta_{i}}^{\left(i\right)}{;g}^{*}\right)$

18:      $M_{\Psi }^{g*}\left(\Theta ,i\right)\leftarrow E_{\theta_{i}}\left[M_{\Psi }^{g*}\left(\Theta_{i,\theta_{i}}\right)\right]$

19: $i^{*}\leftarrow \underset{i=1,2,...,k}{\text{arg}\;\text{min}}\;M_{\Psi }^{g*}\left(\Theta ,i\right)$

20: **return ***i^∗^*

After removing gene *g^∗^*, we find the expected remaining MOCU corresponding to each experiment using equation (6) by replacing $\psi \left(\theta_{\theta_{i}}^{\left(i\right)}\right)$ with $\psi^{ind}\left(\theta_{\theta_{i}}^{\left(i\right)}{;g}^{*}\right)$ and $\psi_{{}_{IBR}}\left({\Theta_{i,\theta }}_{{}_{i}}\right)$ with $\psi_{{}_{{}_{IBR}}}^{ind}\left({\Theta_{i,\theta }}_{{}_{i}};g^{*}\right)$. An abstract form of the proposed experimental design method has been given in Algorithm 1. A step by step toy example illustrating Algorithm 1 is also provided in the Additional file 1.

This procedure for estimating optimal experiment via deleting one gene can be easily extended to the deletion of two or more genes. For example, to delete two genes, we need to evaluate the cost function in (14) for all possible two-gene combinations and delete the pair whose cost is minimum.

#### Reduction mappings and induced interventions

If we want to delete gene *g *from network, we need to find the regulatory function **F***_red _*for the reduced network. Following [13], every two states of the original network that differ only in the value of gene *g *can be collapsed to find the transition rule of the reduced network. Let $s_{1}^{g}$ and $s_{0}^{g}$ be two states with value 1 and 0 for gene *g*, respectively, and identical values for other genes. State $\bar{s^{g}}$ can be obtained from either $s_{1}^{g}$ or $s_{0}^{g}$ by removing the value of gene *g*. If for the original network, the transition rules for these two states are $F\text{(}s_{1}^{g}\text{)}\;\text{ = }\;p$ and $F\text{(}s_{0}^{g}\text{)}\;\text{ = }\;q,$ then for the reduced network, $F_{red}\left(\bar{s^{g}}\right)=\bar{p^{g}}$ if $\pi_{s_{1}^{g}}>\pi_{s_{0}^{g}}$ and otherwise $F_{red}\left(\bar{s^{g}}\right)=\bar{q^{g}}$, where $\bar{p^{g}}$ and $\bar{q^{g}}$ are found from states *p *and *q *via removing the value of gene *g*, respectively. Following this procedure, we find the regulatory function **F***_red _*for all states in the reduced network.

**Algorithm 2 **Finding induced optimal interventions

1: **input**: $\psi \left(\theta^{g}\right)=\left(\hat{u},\hat{v}\right),\pi$

2: **output**: $\psi^{ind}\left(\theta ;g\right)=\left(u_{g}^{ind},v_{g}^{ind}\right)$

3: ${\tilde{u}}_{1}^{g}$*← *place 1 in the *g*th coordinate of $\tilde{u}$

4: ${\tilde{u}}_{0}^{g}$*← *place 0 in the *g*th coordinate of $\tilde{u}$

5: **if $\pi_{{\tilde{u}}_{1}^{g}}\geq \pi_{{\tilde{u}}_{0}^{g}}$ then**

6:   $u_{g}^{ind}\leftarrow {\tilde{u}}_{1}^{g}$

7: **else**

8:   $u_{g}^{ind}\leftarrow {\tilde{u}}_{0}^{g}$

9: ${\tilde{\upsilon }}_{1}^{g}$*← *place 1 in the *g*th coordinate of $\hat{\upsilon }$

10: ${\tilde{\upsilon }}_{0}^{g}$*← *place 0 in the *g*th coordinate of $\hat{\upsilon }$

11: **if **$\pi_{{\hat{v}}_{1}^{g}}\geq \pi_{{\hat{v}}_{0}^{g}}$** then**

12:   $\upsilon_{g}^{ind}\leftarrow {\tilde{\upsilon }}_{1}^{g}$

13: **else**

14:   $\upsilon_{g}^{ind}\leftarrow {\tilde{\upsilon }}_{0}^{g}$

As illustrated in Algorithm 2, we find the induced optimal intervention from the optimal intervention for the reduced network. Suppose that the optimal intervention for the reduced network *θ^g ^*is $\psi \left(\theta^{g}\right)=\left(\hat{u},\hat{v}\right).$ The two corresponding states to $\hat{u}$ in the original network are ${\tilde{u}}_{1}^{g}$ and ${\tilde{u}}_{0}^{g}$, which are found by placing 1 and 0 in the *g*th coordinate of $\hat{u}$, respectively. Similarly, there are two states ${\tilde{v}}_{1}^{g}$ and ${\tilde{v}}_{0}^{g}$ in the original network corresponding to state $\hat{\upsilon }$ The induced optimal intervention for the original network is $\psi^{ind}\left(\theta ;g\right)=\left(u_{g}^{ind},v_{g}^{ind}\right),$ where $u_{g}^{ind}$ is the one among ${\tilde{u}}_{1}^{g}$ and ${\tilde{u}}_{0}^{g}$ having larger steady-state probability in the original network and $v_{g}^{ind}$ is the one among ${\tilde{\upsilon }}_{1}^{g}$ and ${\tilde{\upsilon }}_{0}^{g}$ with larger steady-state probability in the original network.

Analogous to the induced optimal intervention, the induced robust intervention $\psi_{{}_{{}_{IBR}}}^{ind}\left(\Theta ;g\right)$ is found from the robust intervention $\psi_{{}_{{}_{IBR}}}\left(\Theta^{g}\right)$ according to Algorithm 3; however, here we choose the two states possessing larger expected steady-state probability across Θ using the expected SSD, **π**(Θ) = *E_θ _*[**π**(*θ*)], where **π**(*θ*) is the SSD of the network with uncertainty vector *θ *in uncertainty class Θ. We can use this procedure to find the induced robust intervention for each remaining uncertainty class Θ*_i,ϕ_*.

**Algorithm 3 **Finding induced robust interventions

1: **input**: $\psi_{IBR}\left(\Theta^{g}\right)=\left(\hat{u},\hat{\upsilon }\right),\pi \left(\theta \right)\forall \theta \in \Theta$

2: **output**: $\psi_{IBR}^{ind}\left(\Theta ;g\right)=\left(u_{g}^{ind},\upsilon_{g}^{ind}\right)$

3: $\pi \leftarrow E_{\theta }\left[\pi \left(\theta \right)\right]$

4: ${\tilde{u}}_{1}^{g}$*← *placing 1 in the *g*th coordinate of $\hat{u}$

5: ${\tilde{u}}_{0}^{g}$*← *placing 0 in the *g*th coordinate of $\hat{u}$

6: **if **$\pi_{{\tilde{u}}_{1}^{g}}\geq \pi_{{\tilde{u}}_{0}^{g}}$** then**

7:   $u_{g}^{ind}\leftarrow {\tilde{u}}_{1}^{g}$

8: **else**

9:   $u_{g}^{ind}\leftarrow {\tilde{u}}_{0}^{g}$

10: ${\tilde{v}}_{1}^{g}$*← *placing 1 in the *g*th coordinate of $\hat{\upsilon }$

11: ${\tilde{v}}_{0}^{g}$*← *placing 0 in the *g*th coordinate of $\hat{\upsilon }$

12: **if **$\pi_{{\tilde{\upsilon }}_{1}^{g}}\geq \pi_{{\tilde{\upsilon }}_{0}^{g}}$** then**

13:   $\upsilon_{g}^{ind}\leftarrow {\tilde{\upsilon }}_{1}^{g}$

14: **else**

15:   $\upsilon_{g}^{ind}\leftarrow {\tilde{\upsilon }}_{0}^{g}$

#### Preliminary gene elimination via the coefficient of determination

To further reduce the computational cost of the experimental design, we utilize the coefficient of determination (CoD) [27] to eliminate some genes from the optimization problem without evaluating the cost function and then search among the remaining genes for choosing genes to be removed using the cost function (14). The CoD measures the strength of relationship between a target gene *Y *and a vector *X *of predictor genes as the difference between the error of the best estimation of gene *Y *in the absence of other genes and in the presence of genes in *X*. The CoD is between 0 and 1 and a larger CoD means a stronger connection between the target and predictor genes, in our case the target gene being the aim of intervention. We use the intuition that genes possessing large CoD in relation to the target gene are not likely among the genes that should be deleted because they have strong connection to the target gene. The CoD of the target gene *Y*, relative to a vector *X *= (*X*_1_*,..., X_m_*) of predictor genes is defined by

(15) $$CoD_{X}\left(Y\right)=\frac{\varepsilon_{Y}-\varepsilon_{X,Y}}{\varepsilon_{Y}}$$

where *ε_Y _*is the error of the best estimation of *Y *without any predictors,

(16) $$\varepsilon_{Y}=\text{min}\left[P\left(Y=0\right),P\left(Y=1\right)\right],$$

and $\varepsilon_{X,Y}$ is the error of the optimal estimation of *Y *upon observing *X*. By assuming that the value of the binary vector *X *of predictor genes changes from 1 to 2*^m^*, *ε_X,Y _*can be calculated by

(17) $$\begin{array}{c} \varepsilon_{X,Y}=\\ \overset{2^{m}}{\underset{j=1}{\sum }}P\left(X=j\right)\text{min}\left[P\left(Y=0|X=j\right),P\left(Y=1|X=j\right)\right]. \end{array}$$

If *CoD_X_*(*Y *; *θ*) denotes the CoD of *Y *relative to *X *in a network with uncertainty vector *θ*, then given the uncertainty class Θ the expected CoD of *Y *relative to *X *is given by

(18) $$CoD_{X}\left(Y;\Theta \right)=E_{\theta }\left[CoD_{X}\left(Y;\theta \right)\right].$$

Genes possessing strong connection with the target gene in terms of *CoD_X_*(*Y *; Θ) are not considered for deletion. When excluding genes using the CoD it is important to recognize the possibility of intrinsic multivariate prediction [28], where a set of genes may have low individual CoDs with respect to the target gene but may have significant CoD when used together for multivariate prediction. First we calculate *CoD_X_*(*Y *; Θ) for all 3-gene combinations and pick the one with largest CoD. We compute CoD for 3-gene predictors because it has been shown in [17] that the average connectivity of the model cannot be too high providing that the model is not chaotic and it is commonplace to assume 3-gene predictivity in BNs. If we want to exclude less than 3 genes from the search space, then among the 3-gene combination with the largest expected CoD, we choose those genes that have larger expected individual CoD. If we want to exclude more than 3 genes, then in addition to the three genes in the combination with the largest CoD, we choose those genes in the 3-gene combination with the second largest CoD that have larger expected individual CoD and do not belong to the first 3-gene combination. We repeat this process until we reach the desired number of genes to exclude.

If there are initially *n *genes and we want to delete 3 genes, then we need to evaluate cost function (14) for all *C*(*n*, 3) 3-gene combinations, where *C*(*n, k*) denotes the number of combinations of *n *objects taken *k *at a time; however, if we exclude *s *genes from search space then the number of evaluations of (14) decreases to *C*(*n *− *s*, 3).

Having performed the CoD-based exclusion process and excluded *s *genes, $X_{1}^{\prime },X_{2}^{\prime },...,X_{s}^{\prime },$ we search for the genes to be deleted using the cost function in (14) among the remaining genes, {*X*_1_, *X*_2_,..., *X_n_*} − $\left\{X_{1}^{\prime },X_{2}^{\prime },...,X_{s}^{\prime }\right\}$.

### Computational complexity analysis

The first step for the optimal experimental design in [10] is estimating optimal interventions ψ(θ) for each network in Θ. We also need to compute the robust intervention $\psi_{IBR}\left(\Theta_{i,\theta_{i}}\right)$ for each possible remaining uncertainty class Θ*_i,θi_*. Most of the computations are devoted to this step. Finding robust interventions does not require additional calculations because we can store the error of each intervention ψ∈Ψ for the network *θ *when finding optimal interventions and later use these errors to find robust interventions. Therefore, complexity analysis requires computing the complexity of estimating the optimal interventions.

With *n *genes that take on binary expression levels, the network has 2*^n ^*states. Finding an optimal single-gene function intervention requires searching among all possible 2^2*n *^state pairs (*u, v*) according to (1). Assuming without loss of generality that states 2^*n*−1 ^to 2*^n ^*are undesirable, (1) must be evaluated 2^*n*−1 ^times for each state pair. Thus, the complexity of finding the optimal intervention *ψ*(*θ*) is  O(2^3*n*^). If there are *k *uncertain parameters and each can take on *l *different values, then the uncertainty class Θ contains *l^k ^*different networks for which an optimal intervention must be found. Hence, the complexity of the optimal experimental design method in [10] is  O(*l^k ^*× 2^3*n*^).

To analyze the complexity of the proposed approximate method, suppose *p *genes are to be deleted. Then the cost function in (14) must be evaluated for all *C*(*n *− 1, *p*) *p*-gene combinations, *n *− 1 instead of *n be*cause the target gene cannot be deleted. The complexity of finding an induced optimal intervention for each network after deleting *p *genes is  O(2^3(*n*−*p*)^). Therefore, the complexity of the approximate method is  O(*C*(*n *− 1, *p*) × *l^k ^*× 2^3*n*−3*p*^). For large *n*, it is possible that for small *p *the complexity of the approximate method can exceed that of the original method; however, by deleting more genes the complexity of the approximate method drops sharply because by deleting each additional gene the complexity of estimating the optimal intervention decreases by eight-fold.

By incorporating the CoD-based gene exclusion step in the approximation method and excluding *s *genes we are able to decrease the number of *p*-gene combinations from *C*(*n *− 1, *p*) to *C*(*n *− *s *− 1, *p*), which reduces the complexity of the approximate method to  O(*C*(*n*−*s*− 1,*p*) × *l^k ^*× 2^3*n*−3*p*^). Define the computational gain *λ *by

(19) $$\lambda =\frac{l^{k}\times 2^{3n}}{C\left(n-s-1,p\right)\times l^{k}\times 2^{3n-3p}}=\frac{2^{3p}}{C\left(n-s-1,p\right)},$$

which is the ratio of the complexity of the optimal method in [10] to the complexity of the approximate method when deleting *p *genes using the cost function in (14) and excluding *s *genes from the search space using the CoD-based gene exclusion step.

**Figure 2:**
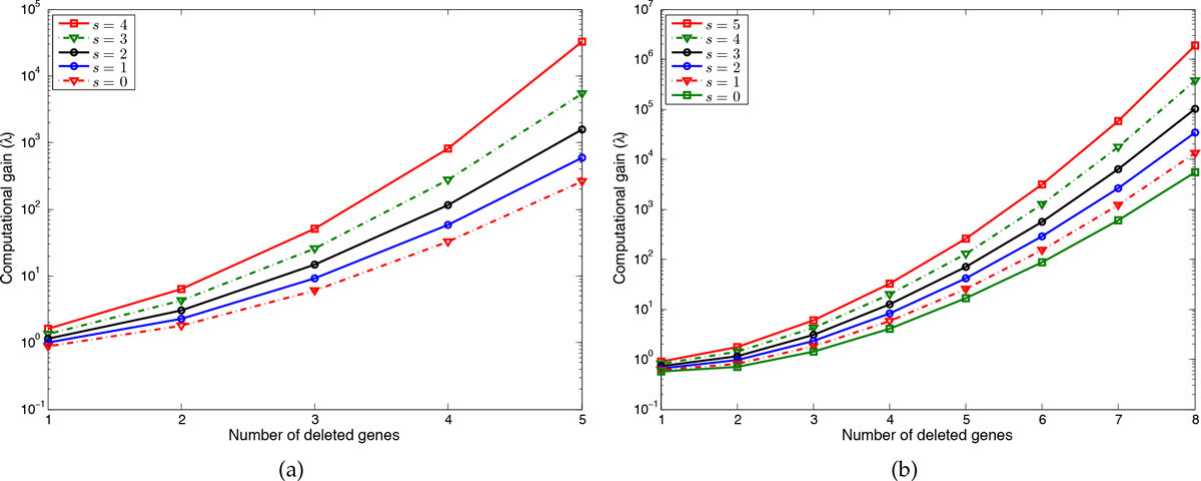
**Computational gain (*λ*) of using the proposed approximate method**. Different number of genes are deleted and *s *genes are excluded from the search space. (a) Networks with *n *= 10 genes. (b) Networks with *n *= 15 genes.

Figure 2 shows the computational gain *λ *when deleting *p *genes and excluding *s *genes from the search space for network size of 10 and 15. Note that for large *n*, if we delete very few genes the complexity might exceed that of method in [10] but as more genes are deleted the complexity of the approximate method becomes much smaller. For example when *n *= 15, searching over all genes and deleting 1, 2, and 3 genes, *λ *= 0.5741, *λ *= 0.7, and *λ *= 1.4, respectively, but for *p *> 3, *λ *grows rapidly, reaching *λ *≈ 600 when deleting 7 genes. Greater computational gain results from excluding some genes using the CoD-based step. For instance, excluding 3 genes from the search space results in *λ *= 1.16 and *λ *≈ 6350 when deleting 2 and 7 genes, respectively.

Table 1 shows the approximate processing times for performing the optimal and proposed experimental design methods for networks of different size with 4 unknown regulations. Simulations have been run on a machine with 8 GB of RAM and Intel(R) Core(TM) i7 CPU, 3.1 GHz. The run times grow exponentially as the number of genes increases. This table clearly suggests that the optimal experimental design method can be applicable to networks of at most *n *= 11 genes but using the proposed approximate experimental design method we can still increase the number of genes in the network. For example, for *n *= 12 genes, optimal experimental design takes around 17 hours to complete but when we use the proposed method and delete 5 genes it takes around 8 minutes without gene exclusion and 3 minutes with 2-gene exclusion - a significant saving in processing time. Note that the ratios between times in Table 1 do not exactly follow the computational gain in (19), especially when the size of the reduced network is very small, because the times in the table also include the time required for calculating the SSD, TPM, and fundamental matrices for original networks in Θ.

**Table 1:** Comparing the approximate processing times (in seconds) of the optimal and approximate experimental design methods when *p *genes are deleted and *s *genes are excluded for networks of size *n *with 4 uncertain regulations

	*n *= 10		*n *= 11		*n *= 12	
	**468**		**4846**		**60169**	

Proposed	*s *= 0	*s *= 2	*s *= 0	*s *= 2	*s *= 0	*s *= 2
			
*p *= 3	81.71	39.64	830	407	9795	5026
*p *= 4	23.61	12.98	215	93	2355	1057
*p *= 5	8.68	8.36	58	35	450	181

## Results and discussion

This section evaluates the performance of the approximate method for both synthetic and real GRNs where the majority vote rule is used as the transition rule. Majority vote rule [29-33] is popular in systems biology, especially when we are interested in the overall dynamics of the network. For example, majority vote is used in [32] to model the dynamics of yeast cell-cycle network. For the majority vote rule, a regulatory matrix **R **is defined component-wise by

$R_{ij}=\left\{\begin{array}{cc} 1 & \text{gene}\;j\;\text{activates}\;\text{gene}\;i\\ -1 & \text{gene}\;j\;\text{suppresses}\;\text{gene}\;i\\ 0 & \text{no}\;\text{relation}\;\text{from}\;\text{gene}\;j\;\text{to}\;\text{gene}\;i \end{array}\right).$

According to this rule, gene *i *takes value 1 if the number of genes that are ON and activate it is more than the number of genes that are ON and suppress it:

$$\begin{array}{c} X_{i}\left(t+1\right)=f_{i}\left(X\left(t\right)\right)\\ =\left\{\begin{array}{cc} 1 & \text{if}\;\sum_{j}R_{ij}X_{j}\left(t\right)>0\\ 0 & \text{if}\;\sum_{j}R_{ij}X_{j}\left(t\right)<0\\ X_{i}\left(t\right) & \text{if}\;\sum_{j}R_{ij}X_{j}\left(t\right)=0 \end{array}.\right) \end{array}$$

Uncertainty is introduced by assuming that the exact values of some of the nonzero components of **R **are unknown; that is, for some regulations it is not known whether they are activating or suppressive. Each uncertain parameter *θ*_*i *_can be −1 or 1. Conducting experiment *E_i _*determines the value of parameter *θ*_*i*_. Let *μ *= (*μ*_1_,..., *μ_k_*) denote the true value for the uncertainty vector *θ *= (*θ*_1_,*θ*_2_,..., *θ_k_*). Conducting experiment *E_i _*results in a remaining uncertainty class $\Theta_{i,\mu_{i}}$ consisting of networks with *θ_i _*= *μ_i _*and other uncertain parameters being −1 or 1. For $\Theta_{i,\mu_{i}}$ we can determine a robust intervention $\psi_{IBR}\left(\Theta_{i,\mu_{i}}\right)$. We evaluate the effectiveness of experiment *E_i _*in terms of the error of the resulting robust intervention obtained after experiment on the underlying true network, $\xi_{\mu }\left(\psi_{IBR}\left(\Theta_{i,\mu_{i}}\right)\right)$. We define the gain of conducting the chosen experiment E_i* _over a random experiment *E_rnd _*(chosen randomly without using any experimental design) by

(20) $$\rho =\xi_{\mu }\left(\psi_{IBR}\left(\Theta_{rnd,\mu_{rnd}}\right)\right)-\xi_{\mu }\left(\psi_{IBR}\left(\Theta_{i*,\mu_{i*}}\right)\right).$$

If *ρ *> 0, then the chosen experiment outperforms the random experiment; if *ρ *< 0, then the random experiment outperforms the chosen experiment; and if *ρ *= 0, then they perform the same.

### Simulation results based on the synthetic BNps

For the performance evaluation based on synthetic BNps, we generated 1000 networks randomly and chose 50 different sets of *k *regulations in each to be unknown - 50000 simulations in total. We assigned 3 random predictor genes to each gene where each one can be randomly activating or suppressive. The gene perturbation probability was set to 0.001. Without loss of generality, we assume that states with up-regulated *X*_1 _are undesirable. We removed the regulatory type of those regulations that have been assumed to be uncertain and retained other regulatory information of the network. We assume that all uncertain parameters are independent from each other and have uniform marginal distribution. The analysis can be easily extended to other distributions. Because *X*_1 _is the target gene, it was excluded from the reduction process. Hence, we look for the best *p*-gene set to be deleted among {*X*_2_,..., *X_n_*}.

**Figure 3:**
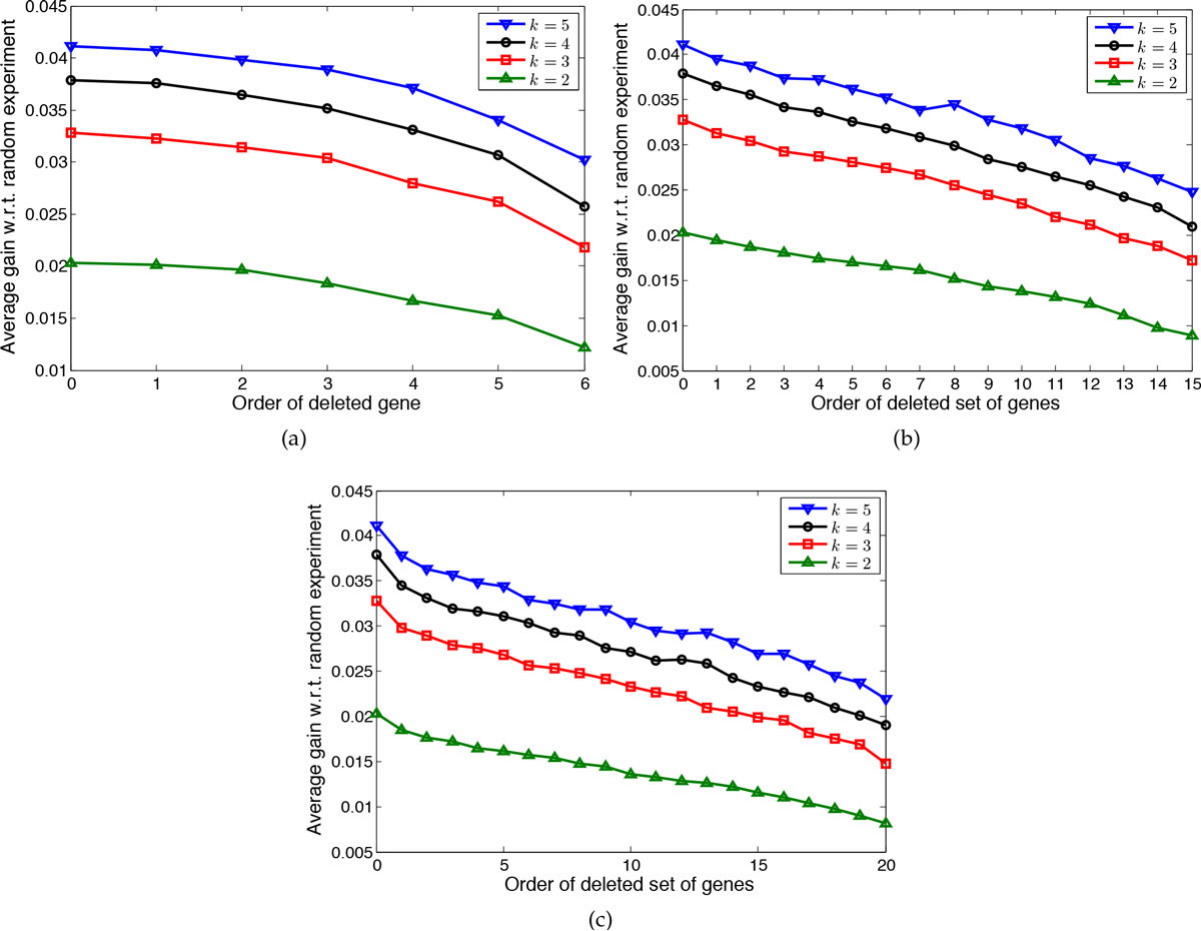
**Evaluating the effectiveness of the proposed cost function for 7-gene networks with *k *uncertain regulations**. The average gain of conducting the chosen experiments by the proposed approximate method with respect to the random experiments when deleting different genes is shown. (a) Deleting one gene. (b) Deleting two genes. (c) Deleting three genes.

Figure 3 shows the average gain *ρ *for networks with *n *= 7 genes and *k = *2, 3, 4, 5 uncertain regulations. For each *k*, we delete 1, 2, and 3 genes. Given the deletion of *p *genes, to evaluate the effectiveness of the proposed cost function in (14), we rank all *p*-gene combinations based on this cost function and compare the performance of the proposed approximate method when deleting each of these sets. For example in Figure 3(a), there are 6 different choices for a single gene to be deleted or in Figure 3(b) there are *C*(6, 2) = 15 different selections for two genes to be deleted. In all subfigures in 3, the average gain when the order of the deleted set is 0 corresponds to optimal experimental design [10]. This figure shows that for different number of uncertain regulations and different number of deleted genes, deleting those sets that correspond to a lower cost function results in larger average *ρ*. Denoting average *ρ *by $\bar{\rho }$, for *k *= 5, where $\bar{\rho }=0.0\text{411}$ for the optimal method, if we delete the gene with minimum cost, then $\bar{\rho }=0.0\text{4}0\text{8}$, but if we delete the gene with maximum cost, then $\bar{\rho }=0.0\text{3}0\text{2}$. When deleting two genes, corresponding to the best pair of genes (corresponding to the minimum cost) $\bar{\rho }=0.0\text{395}$ but for the pair corresponding to the largest cost (15th set) $\bar{\rho }=\;0.0\text{248}$. When deleting three genes, for the best set of deleted genes $\bar{\rho }=0.0\text{378}$ and for the worst set $\bar{\rho }=0.0\text{219}$.

**Figure 4:**
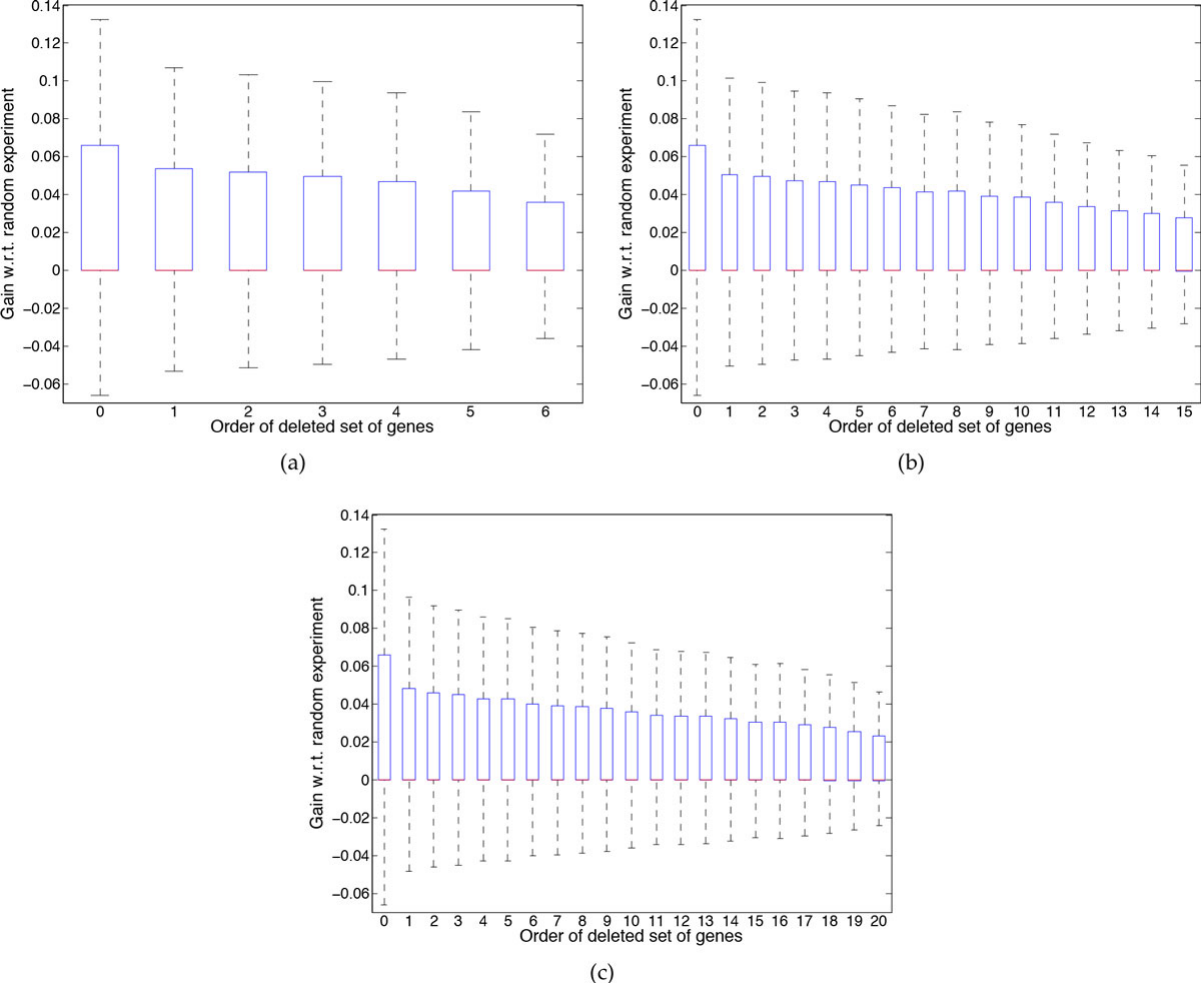
**The box plot of the gain of conducting the chosen experiment by the proposed approximate method with respect to the random experiment when deleting different genes**. 7-gene networks with 5 uncertain regulations are considered. (a) Deleting one gene. (b) Deleting two genes. (c) Deleting three genes.

Figure 4 provides the box plots for 7-gene networks possessing 5 uncertain regulations when 1, 2, and 3 genes are deleted. The box extends from the first quartile (25th percentile) to the third quartile (75th percentile) of the data. The lines extending vertically from the box are called "whiskers". Herein we set whisker length to the interquartile range (distance between the first and third quartiles). The red line in the box represents the median. Note that in the given box plots median and first quartile might not be distinguishable as they are very close to each other but in fact they have different values. The number on the x-axis is the ranking of the set of deleted genes, running from the minimum cost of deletion on the left to the maximum cost of deletion on the right. For optimal experimental design the first quartile, median and third quartile are −1.57 × 10^−5^, 5.38 × 10^−5^, and 0.66172, respectively. For approximate experimental design, as we delete gene(s) whose corresponding cost function is larger, the first quartile, median, and third quartile decrease. For example, in case of deleting 3 genes, if we delete the set of genes corresponding to the minimum cost of deletion the first quartile, median, and third quartile are −3.53 × 10^−5^, 1.51 × 10^−6^, and 0.04816, respectively but if we delete the set of genes with the maximum of deletion cost the first quartile, median, and third quartile would be −0.00055, 0, and 0.022948 respectively. These box plots indicate the promising performance of the proposed cost function because the boxes cover larger values when we delete set of genes possessing smaller cost function.

**Figure 5:**
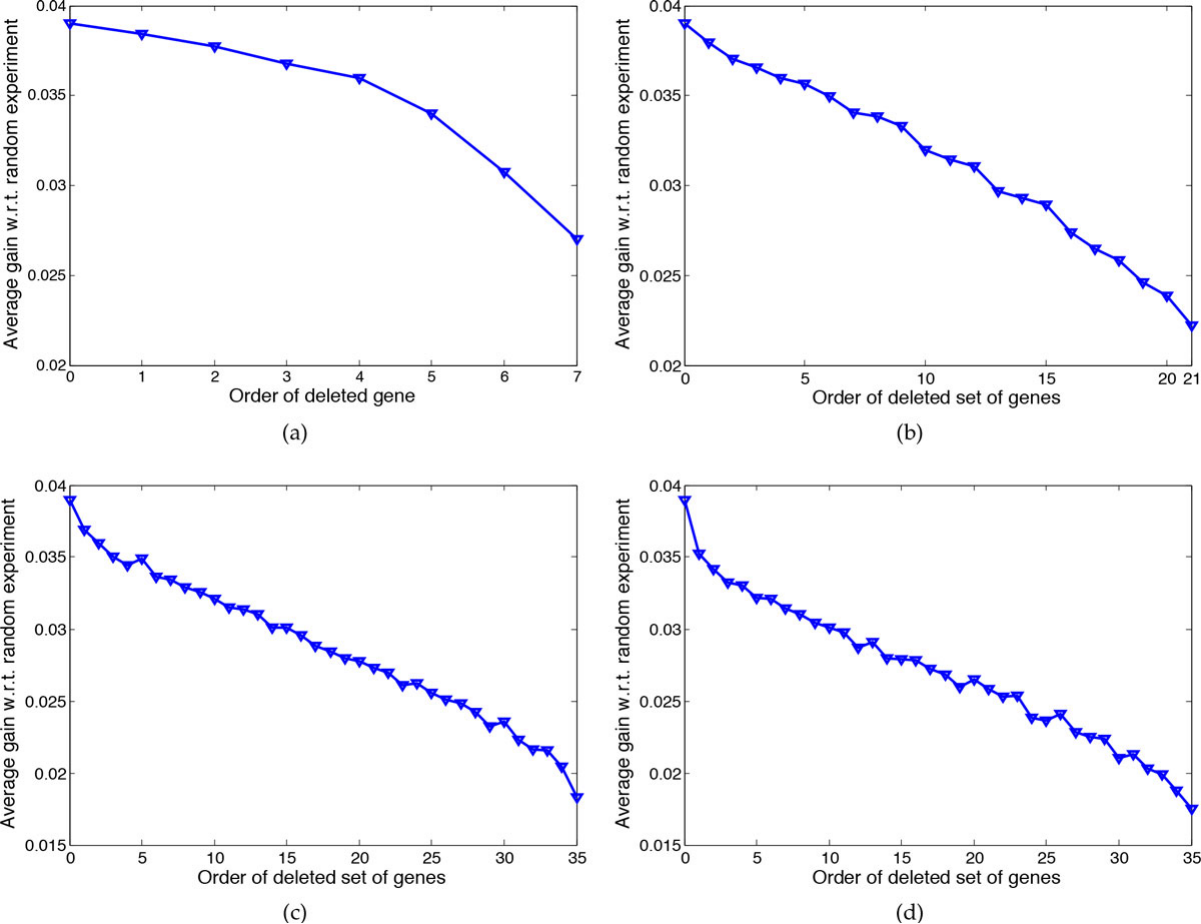
**Evaluating the effectiveness of the proposed cost function for 8-gene networks with 4 uncertain regulations**. The average gain of conducting the chosen experiments by the proposed approximate method with respect to the random experiments is shown. (a) Deleting one gene. (b) Deleting two genes. (c) Deleting three genes. (d) Deleting four genes.

Figure 5 shows performance evaluation for 8-gene networks with *k *= 4 uncertain regulations, deleting up to four genes from the original networks. Again, this figure verifies the promising performance of the proposed cost function. It can be observed that when gene sets possessing larger cost are deleted, the resulting average gain decreases. For example, when we delete 4 genes $\bar{\rho }=0.0\text{39}0$ for the optimal method and $\bar{\rho }$ for the approximate method decreases from 0.0352 to 0.0175 if we delete the 35th set of 4 genes according to the cost function instead of the first set. We also provide the box plots for the performance of the approximate method for 8-gene networks in the Additional file 1.

To consider larger networks, we generated 100 random 13-gene networks and in each chose one set of 4 regulations to be unknown. We used the approximate experimental design method and deleted 5 genes. For this size of network it is not possible to perform the optimal experimental design method or compute original optimal and robust interventions to calculate the gain of the chosen experiment over a randomly selected experiment. Hence, we use the induced robust intervention obtained by deleting the set of 5 genes having the minimum cost of deletion as in (14). Therefore, here gain *ρ *is defined as

$$\rho =\xi_{\mu }\left(\psi_{IBR}^{ind}\left(\Theta_{rnd,\mu_{rnd}}\right);g^{*}\right)-\xi_{\mu }\left(\psi_{IBR}^{ind}\left(\Theta_{i*,\mu_{i*}}\right);g^{*}\right)$$

where g^* ^is the set of 5 genes with minimum cost of deletion. For this set of simulations, the average gain $\bar{\rho }$ is 0.0192. Note that here the average gain might not be very accurate owing to the small number of simulations. The approximate run time for each simulation was around 5700 seconds.

**Figure 6:**
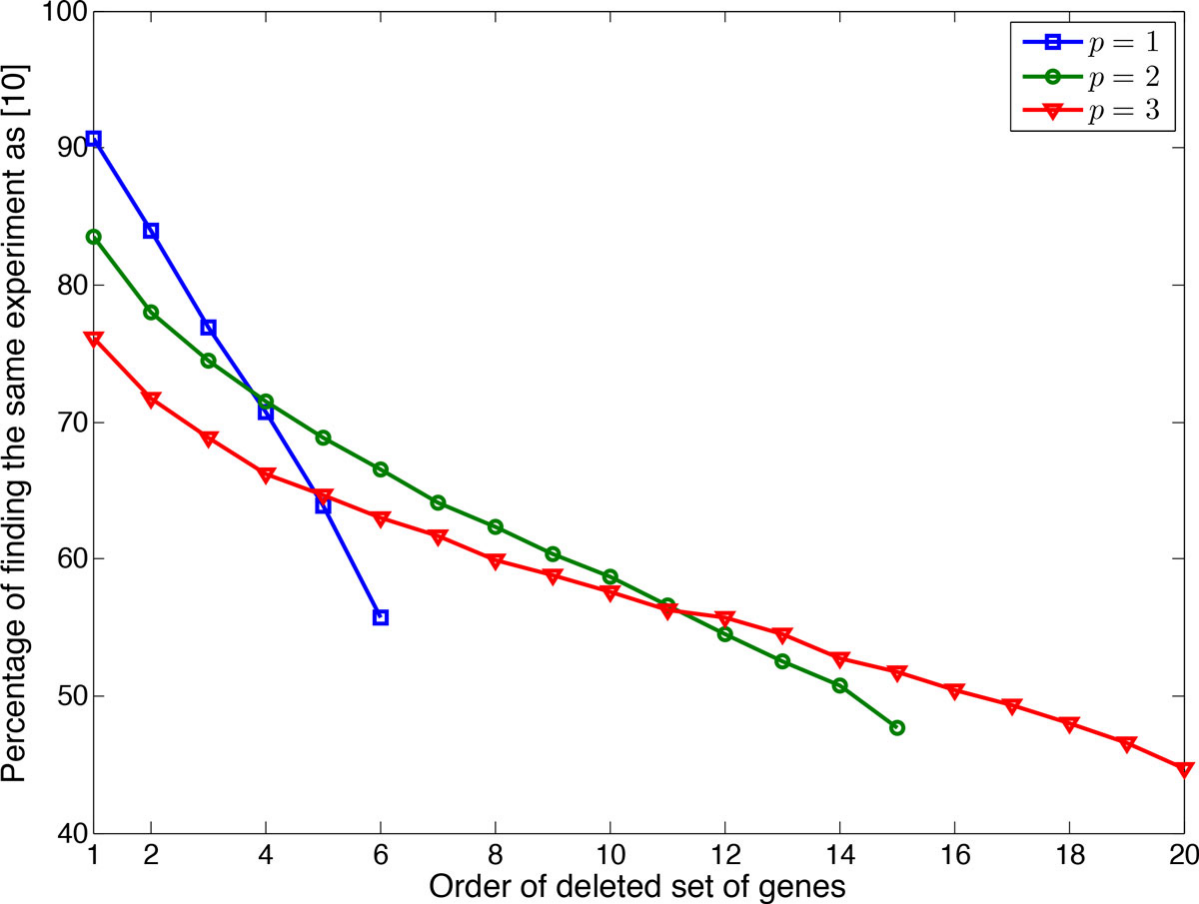
**Percentage of finding the same experiment as the optimal method**. Different *p *genes are deleted for 7-gene networks possessing *k *= 4 uncertain regulations. Gene sets with larger order have higher cost function.

We now evaluate the proportion of times that we obtain the optimal experiment found by [10] when using the approximate method. Figure 6 shows the percentage of finding the optimal experiment when using the approximate method and deleting different number of genes from 7-gene networks. In this figure, there are 6, 15, and 20 values for deleting 1, 2, or 3 genes, respectively. We observe that deleting the set of genes corresponding to the minimum of the cost function yields the highest likelihood of obtaining an optimal experiment, which is what we would hope for from an efficient approximate method. According to Figure 6, when we delete the gene which attains the minimum cost, 90.72% of the simulations yield an optimal experiment, whereas this percentage is 55.73% when deleting the gene with the largest value of the cost function. Similar behavior is observed when deleting 2 or 3 genes. A salient reason that the largest average gain of the approximate method over random experiments is when we delete genes corresponding to the minimum cost function is that it is more likely to get an optimal experiment.

**Figure 7:**
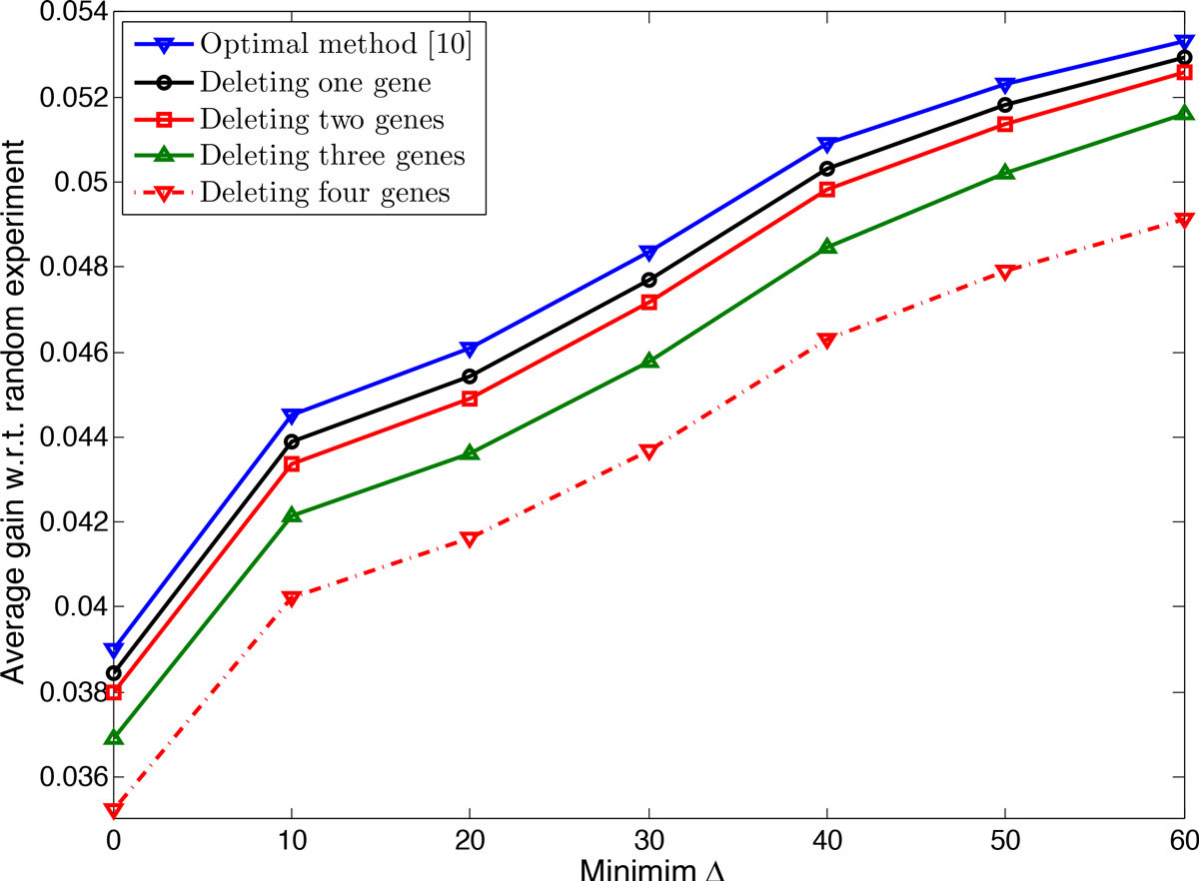
**Effect of controllability Δ on the performance of the experimental design method**. Optimal method in [10] and the proposed approximate method when deleting *p *genes are considered for networks with *n *= 8 genes and *k *= 4 uncertain regulations.

An issue that arises when evaluating experimental design on synthetic networks, as opposed to real biological networks, which typically manifest substantial controllability on account of their need to maintain functionality within changing contexts, a large portion of randomly generated networks might not be controllable and therefore not be responsive to intervention. Hence, intervention has negligible effect on their SSDs and including them in the analysis masks the effect of optimality. To address this issue, we define *controllability *Δ as the percentage decrease of undesirable probability mass after applying optimal intervention:

(21) $$\Delta =\frac{\pi_{U}-{\tilde{\pi }}_{U}}{\pi_{U}}\times \text{1}00\%,$$

where $\pi_{U}$ and ${\tilde{\pi }}_{U}$ are the undesirable probability masses before and after applying optimal intervention to the network, respectively. A larger Δ means that a network is more controllable. Figure 7 considers the effect of controllability on the performance of experimental design when networks have *n *= 8 genes and *k *= 4 uncertain regulations. The figure shows the average gain $\bar{\rho }$ for the optimal method and the proposed approximate method for networks possessing controllability greater than a given threshold. We observe that $\bar{\rho }$ increases when networks are more controllable, regardless of the number of genes deleted from network. Note that as controllability increases, the difference between the performance of different methods increases. For example, for all networks the average gain for the optimal method and the proposed method when deleting one, two, three, and four genes is 0.0390, 0.0384, 0.0380, 0.0369, and 0.0352, respectively; but for networks with Δ ≥ 40% the average gains are 0.0509, 0.0503, 0.0498, 0.0484, and 0.0463, respectively.

**Figure 8:**
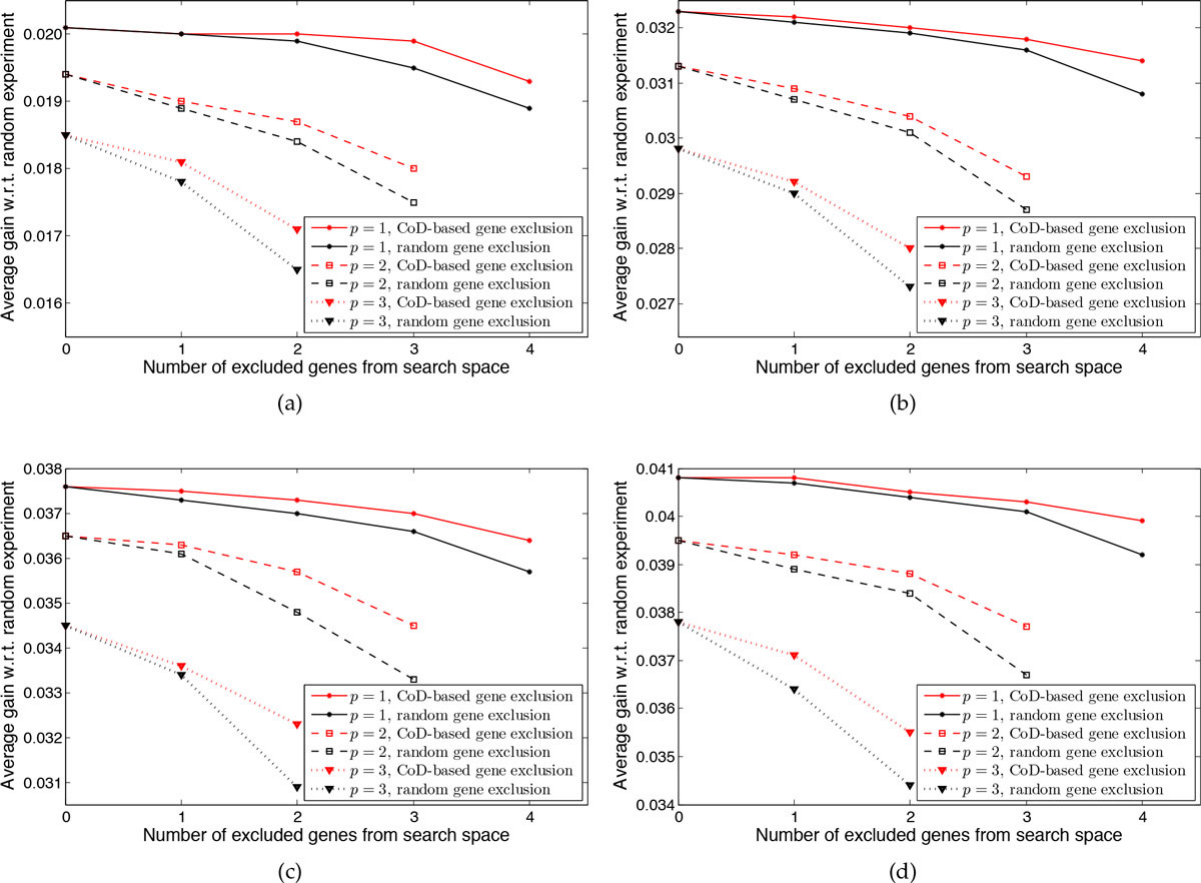
**Performance evaluation of the CoD-based gene exclusion scheme for 7-gene networks**. The average gain of the proposed method over the random experiments when *p *genes are deleted and different number of genes are excluded from the search space is shown. (a) *k *= 2 uncertain regulations. (b) *k *= 3 uncertain regulations. (c) *k *= 4 uncertain regulations. (d) *k *= 5 uncertain regulations.

To evaluate the effectiveness of the CoD-based gene exclusion algorithm, we compare the average gain of the approximate method when excluding genes from the search space using the CoD-based exclusion algorithm against the average gain when excluding randomly selected genes from the search space. Figure 8 shows the average gain $\bar{\rho }$ for networks with *n *= 7 genes and *k * = 2, 3, 4, 5 uncertain regulations. For deleting *p *genes, we exclude up to 6− *p *−1 genes from the search space so that for the largest number of genes excluded, the search space contains at least *p *+ 1 genes. For example, when deleting *p *= 1 gene, we exclude 1, 2, 3, and 4 genes; when deleting *p *= 2 genes we exclude 1, 2, and 3 genes; and so on. For each number of uncertain regulations, we observe that the average gain when excluding genes using the CoD-based algorithm is always larger than random gene exclusion, regardless of the number of deleted genes. For example, when *k *= 5, for deleting one gene and excluding 1, 2, 3, and 4 genes randomly, $\bar{\rho }=0.0\text{4}0\text{7},\;0.0\text{4}0\text{4},\;0.0\text{4}0\text{1}$, and 0.0392 respectively but using the CoD-based scheme and excluding the same number of genes, $\bar{\rho }=0.0\text{4}0\text{8},\;0.0\text{4}0\text{5},\;0.0\text{4}0\text{3}$, and 0.0399 respectively. If we delete three genes $\bar{\rho }=0.0\text{378}$ without gene exclusion, and if we exclude 1 and 2 genes, then $\bar{\rho }=0.0\text{364}$ and $\bar{\rho }=0.0\text{344}$, respectively, when we exclude genes randomly and $\bar{\rho }=0.0\text{371}$ and $\bar{\rho }=0.0\text{355}$, respectively, when we exclude genes based on CoD. Note that when deleting more genes, the difference between random exclusion and CoD-based exclusion increases because as more genes are deleted, exclusion has a larger impact on the number of candidate sets for evaluating the cost function. For example, when deleting 1 gene, if we exclude one gene, then the number of candidate sets decreases from 6 to 5, but when deleting 3 genes, if we exclude one gene, then the number of candidate sets decreases from *C*(6, 3) = 20 to *C*(5, 3) = 10.

**Figure 9:**
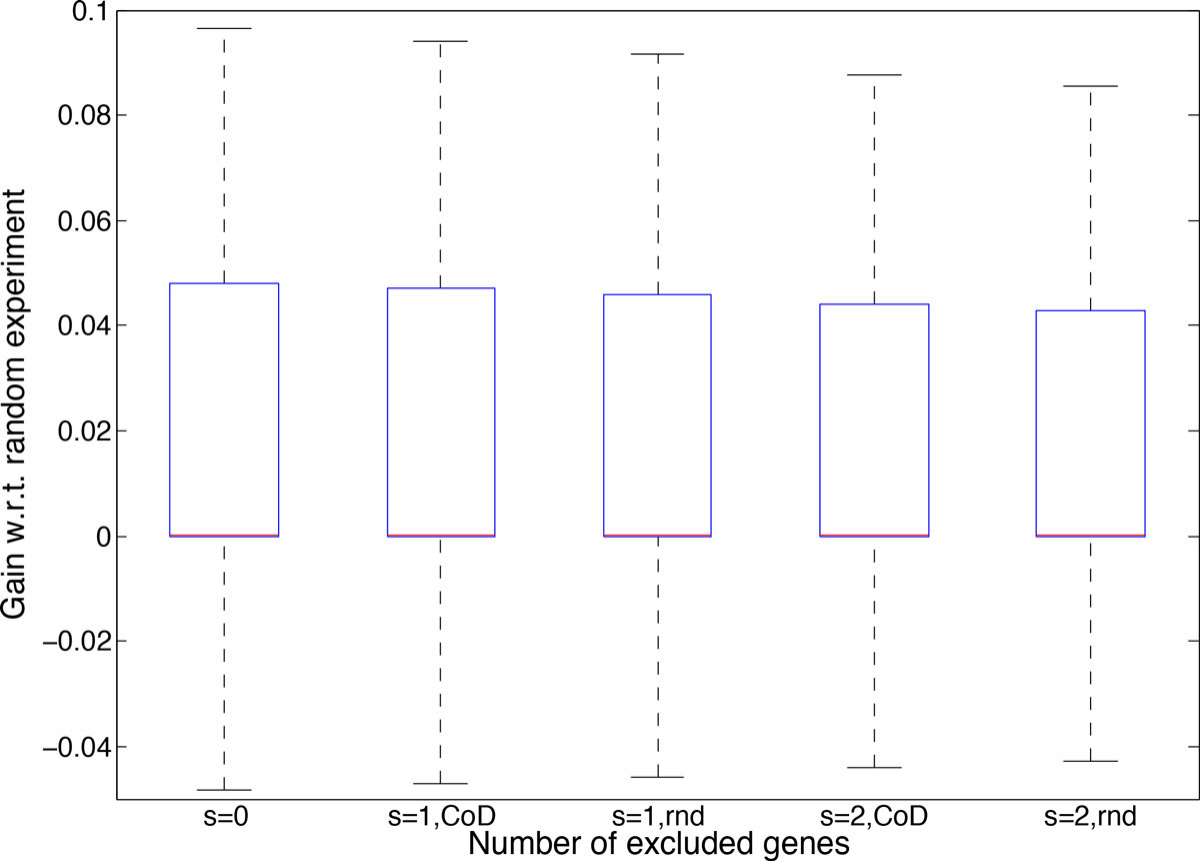
**The box plot of the gain with respect to the random experiment when *s *genes are excluded randomly or using the proposed CoD-based procedure**. 7-gene networks with 5 uncertain regulations are considered.

In Figure 9, we also show the box plot for the gain of conducting the chosen experiment if we delete 3 genes and exclude genes from the search space either randomly or via the proposed CoD-based method for 7 gene networks possessing 5 uncertain regulations. We observe that the first quartile, median, and third quartiles are higher when excluding genes using CoD. For example, when randomly excluding 2 genes from the search space, the first quartile, median, and third quartile are −6.645 × 10^−5^, 3.3289 × 10^−7^, and 0.04274, respectively; however, when excluding genes using CoD they are −5.625 × 10^−5^, 6.044 × 10^−7^, and 0.043907, respectively.

**Figure 10:**
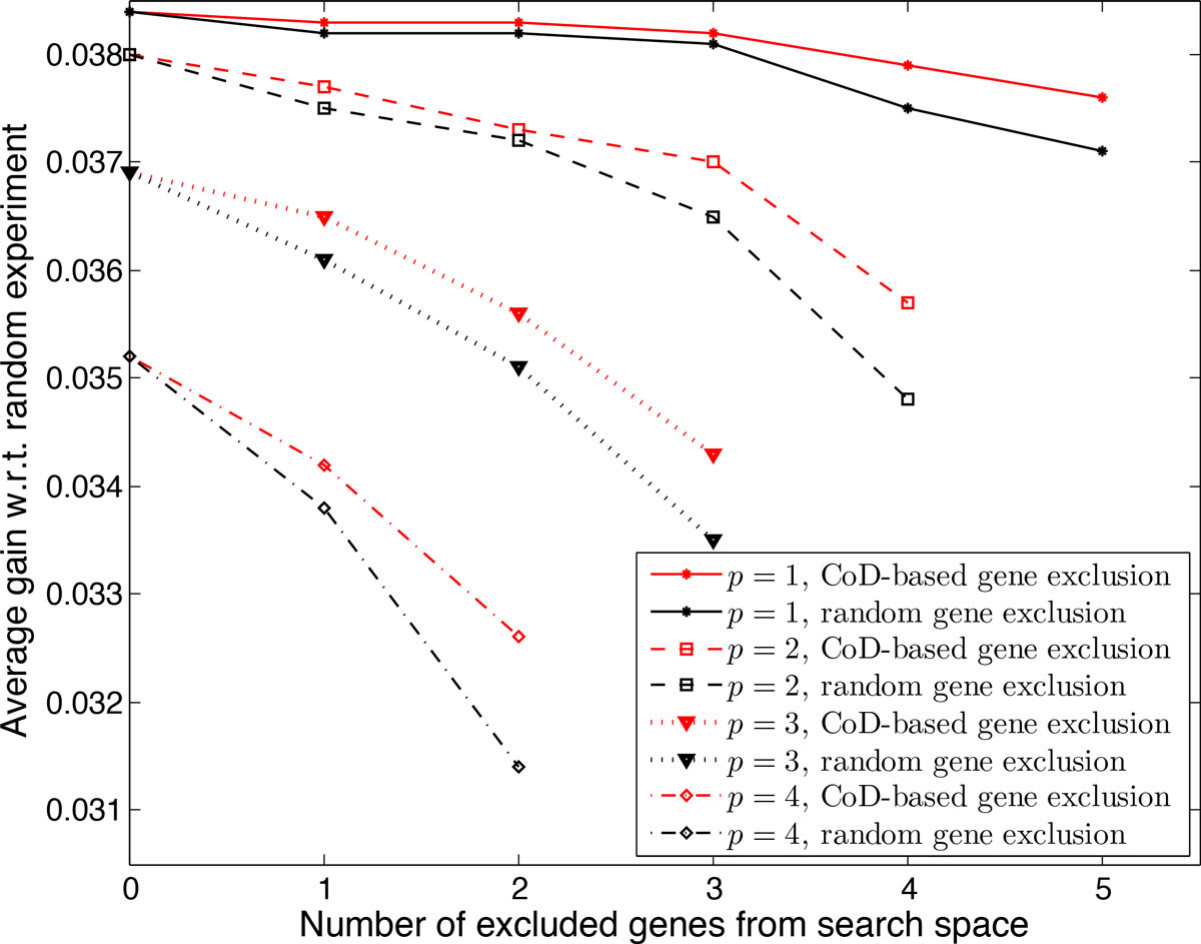
**Performance evaluation of the CoD-based gene exclusion algorithm for 8-gene networks with *k *= 4 uncertain regulations**. The average gain of the proposed approximate experimental design with respect to the random experiments when *p *genes are deleted and different number of genes are excluded from the search space is shown.

Figure 10 is similar to Figure 8 except that it is for 8-gene networks with 4 uncertain regulations. The approximate method is applied deleting 1, 2, 3, and 4 genes. For each number of deleted genes, average *ρ *is computed for random and CoD-based exclusion.

Table 2 lists the percentage that the optimal experiment is found using the approximate method when deleting *p *genes and excluding *s *genes from the search space randomly or according to the CoD-based algorithm. Results are tabulated for 7-gene networks with *k *=2, 3, 4, 5 uncertain regulations and 8-gene networks with *k *= 4 uncertain regulations. Note that if we are interested in deleting *p *genes, *p *+ 1 genes should remain in the search space after the gene exclusion step. For example, for *p *= 2 we exclude up to *s *= 3 genes and for *p *= 3 we exclude up to *s *= 2 genes from the search space. We use N/A in the table for those pairs of *p *and *s *which are not applicable. We observe that the likelihood of obtaining the optimal experiment is larger when we exclude genes according to the CoD-based algorithm rather than excluding them randomly. A larger proportion of experiments found by the approximate method via excluding genes based on CoD agree with the optimal method in [10]. These tables demonstrate the effectiveness of reducing the number of candidate gene sets for the optimization problem by excluding genes based on the CoD.

**Table 2:** Percentage of finding the same experiment as [10] using the proposed approximate method with gene exclusion from the search space

	*n *= 7, *k *= 2							
		*p *= 1		*p *= 2		*p *= 3		
					
		Random	CoD	Random	CoD	Random	CoD	

	*s *= 0	92.59	92.59	87.72	87.72	82.90	82.90	
	*s *= 1	91.78	91.96	85.85	86.44	80.05	80.85	
	*s *= 2	90.23	91.07	83.38	84.77	75.97	77.96	
	*s *= 3	88.31	89.89	79.28	82.01	N/A	N/A	
	*s *= 4	85.18	87.24	N/A	N/A	N/A	N/A	

	*n *= 7, *k *= 3							

		*p *= 1		*p *= 2		*p *= 3		
					
		Random	CoD	Random	CoD	Random	CoD	

	*s *= 0	90.84	90.84	84.19	84.19	77.87	77.87	
	*s *= 1	89.78	90.07	81.94	82.67	74.46	75.49	
	*s *= 2	88.19	89.08	78.67	80.42	69.56	71.58	
	*s *= 3	85.77	87.54	73.95	76.76	N/A	N/A	
	*s *= 4	82.15	84.79	N/A	N/A	N/A	N/A	

	*n *= 7, *k *= 4							

		*p *= 1		*p *= 2		*p *= 3		
					
		Random	CoD	Random	CoD	Random	CoD	

	*s *= 0	90.72	90.72	83.56	83.56	76.17	76.17	
	*s *= 1	89.65	90.06	81.11	82.00	72.44	73.67	
	*s *= 2	87.95	89.08	77.71	79.58	67.10	69.80	
	*s *= 3	85.54	87.57	72.51	76.07	N/A	N/A	
	*s *= 4	81.81	84.51	N/A	N/A	N/A	N/A	

	*n *= 7, *k *= 5							

		*p *= 1		*p *= 2		*p *= 3		
					
		Random	CoD	Random	CoD	Random	CoD	

	*s *= 0	91.28	91.28	83.71	83.71	76.46	76.46	
	*s *= 1	90.21	90.56	81.32	82.31	72.55	73.86	
	*s *= 2	88.60	89.63	78.17	79.96	67.09	69.72	
	*s *= 3	86.33	88.14	72.66	76.26	N/A	N/A	
	*s *= 4	82.64	85.58	N/A	N/A	N/A	N/A	

*n *= 8, *k *= 4								

	*p *= 1		*p *= 2		*p *= 3		*p *= 4	
				
	Random	CoD	Random	CoD	Random	CoD	Random	CoD

*s *= 0	92.43	92.43	86.98	86.98	80.90	80.90	74.97	74.97
*s *= 1	91.69	92.05	85.21	85.94	78.10	79.05	70.95	72.04
*s *= 2	90.59	91.40	82.53	84.13	74.53	76.29	65.81	67.94
*s *= 3	89.03	90.58	79.47	82.00	68.98	71.91	N/A	N/A
*s *= 4	86.83	88.85	74.51	77.93	N/A	N/A	N/A	N/A
*s *= 5	83.09	86.29	N/A	N/A	N/A	N/A	N/A	N/A

**Figure 11:**
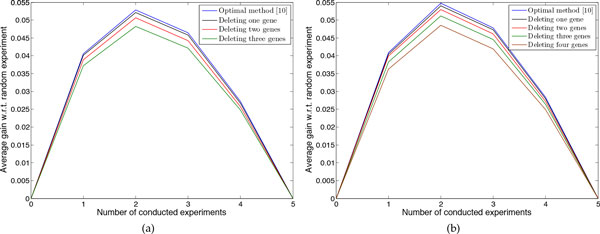
**Performance comparison based on a sequence of experiments**. The average gain for the optimal method in [10] and the proposed approximate method when deleting *p *genes are shown for *k *= 5 uncertain regulations. (a) Networks with *n *= 7 genes. (b) Networks with *n *= 8 genes.

We have also evaluated the performance of the approximate experimental design method when a sequence of experiments is conducted. Suppose there are *k *= 5 uncertain regulations and we conduct five experiments to identify all unknown regulations. For each set of unknown regulations, at each step we utilize the experimental design to choose one of the possible experiments, conduct the chosen experiment, and measure the performance (undesirable probability mass after intervention) of the robust intervention obtained after the experiment on the underlying true network. Continuing, for the remaining uncertain regulations we use the experimental design method and repeat the previous procedure until there is no more unknown regulation remaining in the network. We also do sequential experiments randomly where at each step we choose an experiment randomly, measure the performance of its corresponding robust intervention, and again choose one experiment randomly among the remaining ones. The difference between the undesirable probability mass after applying the robust interventions corresponding to the randomly chosen experiment and the chosen experiment through experimental design at each step is the gain of conducting the chosen experiment at that step. Figure 11 shows the average gain over random selection for the optimal method and the approximate design method deleting up to three genes for 7-gene networks and up to four genes for 8-gene networks. The figure indicates that the approximate design method has reliable performance compared to the optimal method. Moreover, similar to the optimal method, the average gain increases sharply in the beginning for the approximate method. This is very important in real applications owing to the cost and time required for conducting experiments. Note that when we conduct all five experiments the average gain is zero because after five experiments the network is fully known and we can exactly calculate the optimal intervention regardless of the approach taken to choose experiments.

### Performance evaluation based on the colon cancer pathways

**Figure 12:**
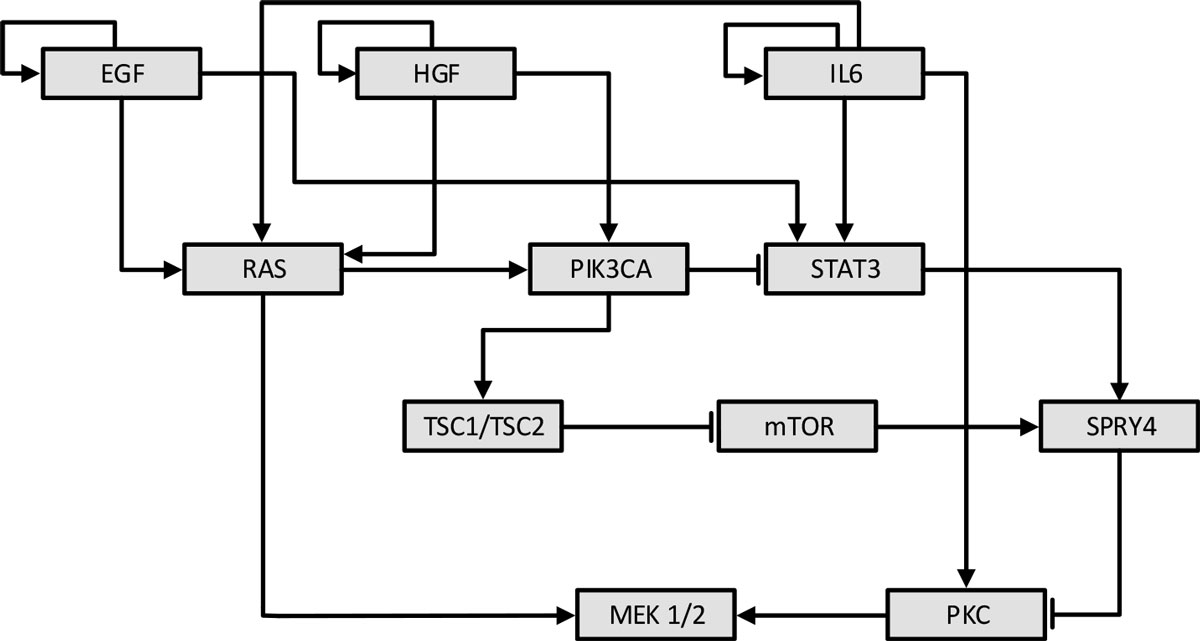
**The regulatory relations among key genes involved in the colon cancer pathways**. Normal arrows represent activating regulations and blunt arrows represent suppressive regulations.

In this section, we analyze the performance of the proposed experimental design method on the colon cancer pathways used in [34]. We focus on the pathways formed by 11 genes extracted from the complete pathway set, as used in [35]. These are shown in Figure 12: *STAT3, RAS, IL6, HGF, PIK3CA, EGF, TSC1/TSC2, mTOR, SPRY4, PKC*, and *MEK 1/2*. Normal and blunt arrows represent activating and suppressive regulations, respectively. We modeled the pathways as a BNp with perturbation probability 0.001. Genes are named as they have been introduced. For example, *STAT3 *is *X*_1 _and *MEK 1/2 *is *X*_11_.

*EGF, HGF*, and *IL6 *are three stimulation factors that carry the external signals generated by neighboring cells to downstream genes and activate downstream pathways. Signal transducers and activators of transcription (*STAT*s) constitute a family of transcription factors that can be activated via extracellular signaling proteins such as cytokins and growth factors. These play a major role in regulating downstream processes such as cell growth, survival, and apoptosis [36]. *STAT3 *is an oncogene observed to be highly activated in many cancers, in particular, colon cancer [37,38]. Hence, *STAT3 *has been recognized as a legitimate target for cancer therapy [39]. We considered states with up-regulated *STAT3 *(*X*_1 _= 1) as undesirable states, so that the set of undesirables states is *U *= {1024,..., 2047}. Before intervention the probability mass of undesirable states $\pi_{U}$ is 0.5525. The optimal intervention for this network is transitioning state 11111110101 to state 01011001101; that is, $\tilde{\text{F}}\left(11111110101\right)=01011001101$ for the regulatory function after intervention. The undesirable probability mass after intervention ${\tilde{\pi }}_{U}$ is 0.3837.

To evaluate the proposed approximate method, we randomly selected 100 different sets of *k *= 4 regulations and assumed that they are uncertain, meaning that their regulatory information is unknown. If experiments are chosen according to the optimal experimental design method in [10], then $\bar{\rho }=0.0\text{244}$. Table 3 compares the average gain $\bar{\rho }$ of the experiments chosen by the approximate method when deleting *p *= 5 genes and excluding *s *genes from the search space using the CoD-based algorithm. The table shows that we can obtain meaningful gain when the approximate experimental design method is used to select the experiment to be conducted first.

**Table 3:** Performance of the proposed approximate method on the colon cancer pathways when deleting *p *genes and excluding *s *genes from the search space

*p *= 3			*p *= 4		*p *= 5	
			
s = 0		s = 2	s = 0	s = 2	s = 0	s = 2
$\bar{\rho }$	0.0239	0.0235	0.0231	0.0229	0.0206	0.0198

## Conclusion

We have proposed a computationally effective experimental design method for reducing uncertainty in gene regulatory networks. This method can effectively approximate the optimal experimental design method in [10], which is based on the mean objective cost of uncertainty (MOCU). To reduce computational complexity, we use network reduction to estimate the optimal and robust interventions needed for finding an optimal experiment. We introduced a novel cost function for gene deletion that takes into account the effect of gene deletion on the ranking of potential experiments. Because potential experiments are ranked based on the MOCU in [10], the proposed cost function is also based on the MOCU. Simulation results on both synthetic and real networks show that while our proposed method can greatly reduce computations, its performance is comparable to the optimal method in [10] and much better than random gene deletion. Greater computational reduction is achieved by excluding genes from the search space based on their CoD with the target gene whose expression the intervention is aimed at altering.

We have assumed a uniform distribution over the uncertainty class. If one has relevant prior knowledge, perhaps it can be used to construct a distribution reflecting it. Care must be taken because concentrating the mass of the distribution in the wrong place can lead to poorer results. In Bayesian terminology, the distribution on the uncertainty class is called a *prior distribution*. Putting a non-uniform prior on Θ does not change the reduction procedure introduced in the current paper; however, some calculations are altered by including the weights. Prior construction is a difficult problem and has been considered in the context of gene regulation, but not in the context of network construction. Rather, pathway knowledge has been used to construct prior distributions governing uncertainty classes of feature-label distributions for optimal Bayesian classification [40,41]. Prior construction is particular to each application, examples being gene/protein signaling pathways in discrete phenotype classification [42] and model-based RNA-seq classification [43]. Prior construction for uncertainty classes of the kind considered in this paper constitutes an important issue for future study - and not just in relation to the specific problem considered herein.

## Competing interests

The authors declare that they have no competing interests.

## Authors' contributions

Conceived the method: RD, BJY, ERD. Developed the algorithm and performed the simulations: RD. Analyzed the results and wrote the paper: RD, BJY, ERD.

## Supplementary Material

Additional file 1 **Supplementary Materials**. This is a file in PDF format that contains an illustrative step by step example providing details about the proposed experimental design method. This file also includes box plots for the simulations on 8-gene networks. Click here for file
