# Erratum to: Efficient experimental design for uncertainty reduction in gene regulatory networks

**DOI:** 10.1186/s12859-015-0839-y

**Published:** 2015-12-14

**Authors:** Roozbeh Dehghannasiri, Byung-Jun Yoon, Edward R. Dougherty

**Affiliations:** Department of Electrical and Computer Engineering, Texas A&M University, College Station, TX 77843 USA; Center for Bioinformatics and Genomic Systems Engineering, Texas A&M University, College Station, TX 77845 USA; College of Science and Engineering, Hamad bin Khalifa University (HBKU), Doha, Qatar

## Erratum

During the production of this article [1], errors occurred in equations and algorithms. The Editorial Department of *BMC Bioinformatics* would like to apologise and inform its readers that an updated version is now available on the *BMC Bioinformatics* website.
