# Supplementary material for: Efficient experimental design for uncertainty reduction in gene regulatory networks
Source: BMC Bioinformatics. 2015 Sep 25;16(Suppl 13):S2. doi: 10.1186/1471-2105-16-S13-S2 (PMC4597030; doi:10.1186/1471-2105-16-S13-S2)
Supplement: Additional file 1 — Supplementary Materials. This is a file in PDF format that contains an illustrative step by step example providing details about the proposed experimental design method. This file also includes box plots for the simulations on 8-gene networks. [file 1471-2105-16-S13-S2-S1.pdf]

Supplementary materials

In this supplemental document we provide an illustrative example to demonstrate the algorithm for the approximate experimental design method proposed by the paper “Efficient experimental design for uncertainty reduction in gene regulatory networks” by Dehghannasiri et al. We also provide the box plots for the results of simulations on 8-gene networks with 4 uncertain regulations.

A. Illustrative example

We consider a 3-gene toy network as shown in Figure 1. This network consists of three genes $\{X_1, X_2, X_3\}$ and three regulations. We assume that the activating regulation from gene X_2 to gene X_3 and the suppressive regulation from gene X_1 to gene X_3 are unknown and denote them by θ_1 and θ_2 respectively. Each uncertain parameter can take two values: 1 for being activating and 2 for being suppressive. Uncertainty class Θ as shown in Figure 2 contains 4 different

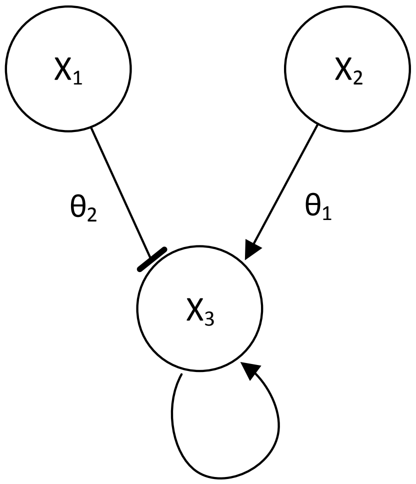

Fig. 1: The 3-gene toy network used for the illustrative example.

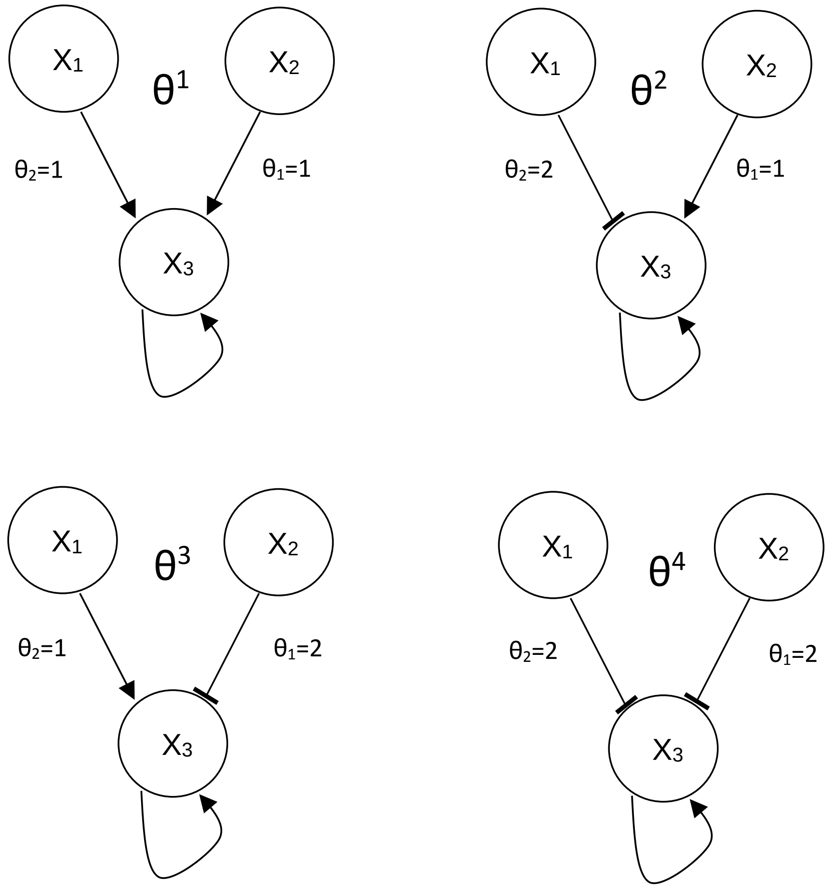

Fig. 2: The uncertainty class Θ which contains all possible networks.

networks: $\{\theta^1, \theta^2, \theta^3, \theta^4\}$ such that:

$$\begin{cases} \theta^1 : (\theta_1 = 1, \theta_2 = 1) \\ \theta^2 : (\theta_1 = 1, \theta_2 = 2) \\ \theta^3 : (\theta_1 = 2, \theta_2 = 1) \\ \theta^4 : (\theta_1 = 2, \theta_2 = 2) \end{cases}$$

Let us assume that the probability density function governing the uncertainty class is uniform and two uncertain parameters are independent from each other. Therefore, all networks within Θ are equally likely having probability $1/4$, i.e., $\mathrm{P}(\theta^i) = 1/4$.

The first step in the proposed experimental design method is to decide which gene is better to be removed. In this example, we assume that the expression state of gene X_3 determines whether a given state is desirable or undesirable. Therefore, we need to find the best gene for

deletion among X_1 and X_2. We go through lines 3 to 12 of Algorithm 1 to calculate the cost of deleting gene X_1 (the sign ✓ is used for comments):

1) Line 3: $g \leftarrow X_1$
2) Line 4: $cost(X_1) \leftarrow 0$
3) Line 5: $i \leftarrow 1$

   ✓ we compute the cost of deleting gene X_1 related to uncertain parameter θ_1.

4) Line 6: $\theta_1 \leftarrow 1$
5) Line 7: $\Theta_{1,1} \leftarrow \{\theta^1, \theta^2\}$, $\Theta_{1,1}^{X_1} \leftarrow \{\theta^{1,X_1}, \theta^{2,X_1}\}$

   ✓ remaining uncertainty class when $\theta_1 = 1$

   ✓ θ^{1,X_1} and θ^{2,X_1} are obtained from θ^1 and θ^2 respectively by deleting gene X_1.

   ✓ we find the reduced networks in $\Theta_{1,1}^{X_1}$ using the procedure given in section "Reduction mappings and induced interventions".

6) Line 8: $\mathrm{P}(\theta^1) \leftarrow 1/4$, $\mathrm{P}(\theta^2) \leftarrow 1/4$

   ✓ probabilities of two networks inside $\Theta_{1,1}$

7) Line 9: $\Psi_{IBR}\left(\Theta_{1,1}^{X_1}\right) \leftarrow \arg\min_{\psi \in \Psi} \left\{\mathrm{P}(\theta^1)\xi_{\theta^{1,X_1}}(\psi) + \mathrm{P}(\theta^2)\xi_{\theta^{2,X_1}}(\psi)\right\}$

   ✓ we found the robust intervention for the uncertainty class $\Theta_{1,1}^{X_1}$ of reduced networks.

   ✓ we can store all costs such as $\xi_{\theta^{1,X_1}}(\psi)$ and $\xi_{\theta^{2,X_1}}(\psi)$ calculated in this step for future computations.

8) Line 9: calculate $\psi_{IBR}^{ind}(\Theta_{1,1}; X_1)$ from $\Psi_{IBR}\left(\Theta_{1,1}^{X_1}\right)$ using Algorithm 3
9) Line 10: $h_{X_1}(\theta_1 = 1) \leftarrow \mathrm{P}(\theta^1)\xi_{\theta^1}\left(\psi_{IBR}^{ind}(\Theta_{1,1}; X_1)\right) + \mathrm{P}(\theta^2)\xi_{\theta^2}; \left(\psi_{IBR}^{ind}(\Theta_{1,1}; X_1)\right)$

   ✓ the average performance of induced intervention across $\Theta_{1,1}$

10) Line 6: $\theta_1 \leftarrow 2$
11) Line 7: $\Theta_{1,2} \leftarrow \{\theta^3, \theta^4\}$, $\Theta_{1,2}^{X_1} \leftarrow \{\theta^{3,X_1}, \theta^{4,X_1}\}$

   ✓ remaining uncertainty class when $\theta_1 = 2$

   ✓ we find the reduced networks in $\Theta_{1,2}^{X_1}$ using the procedure given in section "Reduction mappings and induced interventions".

12) Line 8: $\mathrm{P}(\theta^3) \leftarrow 1/4$, $\mathrm{P}(\theta^4) \leftarrow 1/4$

   ✓ probabilities of two networks inside $\Theta_{1,2}$

13) Line 9: $\Psi_{IBR}(\Theta_{1,2}^{X_1}) \leftarrow \arg\min_{\psi \in \Psi} \left\{\mathrm{P}(\theta^3)\xi_{\theta^{3,X_1}}(\psi) + \mathrm{P}(\theta^4)\xi_{\theta^{4,X_1}}(\psi)\right\}$

- ✓ we found the robust intervention for the uncertainty class $\Theta_{1,2}^{X_1}$ of reduced networks.
- ✓ we can store all costs such as $\xi_{\theta^{3,X_1}}(\psi)$ and $\xi_{\theta^{3,X_1}}(\psi)$ calculated in this step for future computations.

14) Line 9: calculate $\psi_{IBR}^{ind}(\Theta_{1,2}; X_1)$ from $\Psi_{IBR}(\Theta_{1,2}^{X_1})$ using Algorithm 3
15) Line 10: $h_{X_1}(\theta_1 = 2) \leftarrow \mathrm{P}(\theta^3)\xi_{\theta^3}\left(\psi_{IBR}^{ind}(\Theta_{1,2}; X_1)\right) + \mathrm{P}(\theta^4)\xi_{\theta^4};\left(\psi_{IBR}^{ind}(\Theta_{1,2}; X_1)\right)$
16) Line 11: $\mathrm{P}(\theta_1 = 1) \leftarrow 1/2$, $\mathrm{P}(\theta_1 = 2) \leftarrow 1/2$
17) Line 11: $cost(X_1) \leftarrow cost(X_1) + \mathrm{P}(\theta_1 = 1)h_{X_1}(\theta_1 = 1) + \mathrm{P}(\theta_1 = 2)h_{X_1}(\theta_1 = 2)$
    - ✓ we obtained cost for gene X_1 caused by θ_1.
18) Line 5: $i \leftarrow 2$
    - ✓ we now consider uncertain parameter θ_2.
    - ✓ steps 20-31 (for θ_2) are similar to steps 5-16 (for θ_1).
19) Line 6: $\theta_2 \leftarrow 1$
20) Line 7: $\Theta_{2,1} \leftarrow \{\theta^1, \theta^3\}$, $\Theta_{2,1}^{X_1} \leftarrow \{\theta^{1,X_1}, \theta^{3,X_1}\}$
21) Line 8: $\mathrm{P}(\theta^1) \leftarrow 1/4$, $\mathrm{P}(\theta^3) \leftarrow 1/4$
22) Line 9: $\Psi_{IBR}(\Theta_{2,1}^{X_1}) \leftarrow \arg\min_{\psi\in\Psi}\left\{\mathrm{P}(\theta^1)\xi_{\theta^{1,X_1}}(\psi) + \mathrm{P}(\theta^3)\xi_{\theta^{3,X_1}}(\psi)\right\}$
23) Line 9: calculate $\psi_{IBR}^{ind}(\Theta_{2,1}; X_1)$ from $\Psi_{IBR}(\Theta_{2,1}^{X_1})$ using Algorithm 3
24) Line 10: $h_{X_1}(\theta_2 = 1) \leftarrow \mathrm{P}(\theta^1)\xi_{\theta^1}\left(\psi_{IBR}^{ind}(\Theta_{2,1}; X_1)\right) + \mathrm{P}(\theta^3)\xi_{\theta^3};\left(\psi_{IBR}^{ind}(\Theta_{2,1}; X_1)\right)$
25) Line 6: $\theta_2 \leftarrow 2$
26) Line 7: $\Theta_{2,2} \leftarrow \{\theta^2, \theta^4\}$, $\Theta_{2,2}^{X_1} \leftarrow \{\theta^{2,X_1}, \theta^{4,X_1}\}$
27) Line 8: $\mathrm{P}(\theta^2) \leftarrow 1/4$, $\mathrm{P}(\theta^4) \leftarrow 1/4$
28) Line 9: $\Psi_{IBR}(\Theta_{2,2}^{X_1}) \leftarrow \arg\min_{\psi\in\Psi}\left\{\mathrm{P}(\theta^2)\xi_{\theta^{2,X_1}}(\psi) + \mathrm{P}(\theta^4)\xi_{\theta^{4,X_1}}(\psi)\right\}$
29) Line 9: calculate $\psi_{IBR}^{ind}(\Theta_{2,2}; X_1)$ from $\Psi_{IBR}(\Theta_{2,2}^{X_1})$ using Algorithm 3
30) Line 10: $h_{X_1}(\theta_2 = 2) \leftarrow \mathrm{P}(\theta^2)\xi_{\theta^2}\left(\psi_{IBR}^{ind}(\Theta_{2,2}; X_1)\right) + \mathrm{P}(\theta^4)\xi_{\theta^4};\left(\psi_{IBR}^{ind}(\Theta_{2,2}; X_1)\right)$
31) Line 11: $\mathrm{P}(\theta_2 = 1) \leftarrow 1/2$, $\mathrm{P}(\theta_2 = 2) \leftarrow 1/2$
32) Line 11: $cost(X_1) \leftarrow cost(X_1) + \mathrm{P}(\theta_2 = 1)h_{X_1}(\theta_2 = 1) + \mathrm{P}(\theta_2 = 2)h_{X_1}(\theta_2 = 2)$
    - ✓ we found the cost of deleting gene X_1 by adding the cost related to θ_2 to the cost related to θ_1.

At this point we have calculated the cost of deleting gene X_1, $cost(X_1)$. We need to calculate the cost of deleting gene X_2, $cost(X_2)$, as well. The steps for calculating the cost of gene X_2 are similar to those for X_1. Therefore, we skip illustrating these steps and proceed to the next

stage to use the induced optimal and robust interventions found via deleting the optimal gene for the experimental design. Suppose that the optimal gene for deletion is gene X_1 meaning that $cost(X_1) < cost(X_2)$. Therefore, we go through the rest of Algorithm 1 (lines 13 to 19) to estimate the optimal experiment E_{i^*} to be conducted first:

- Line 13: $i \leftarrow 1$
- Line 14: $\theta_1 \leftarrow 1$
- Line 15: $\Theta_{1,1} \leftarrow \{\theta^1, \theta^2\}$
- Line 16: $\mathrm{P}(\theta^1) \leftarrow 1/4$, $\mathrm{P}(\theta^2) \leftarrow 1/4$
- Line 17: $$M_{\Psi}^{X_1}(\Theta_{1,1}) \leftarrow \mathrm{P}(\theta^1)\Big\{\xi_{\theta^1}\left(\psi_{IBR}^{ind}(\Theta_{1,1}; X_1)\right) - \xi_{\theta^1}\left(\psi^{ind}(\theta^1; X_1)\right)\Big\} + \mathrm{P}(\theta^2)\Big\{\xi_{\theta^2}\left(\psi_{IBR}^{ind}(\Theta_{1,1}; X_1)\right) - \xi_{\theta^2}\left(\psi^{ind}(\theta^2; X_1)\right)\Big\}$$
  ✓ we estimated the remaining MOCU when $\theta_1 = 1$ via deleting gene X_1.
- Line 14: $\theta_1 \leftarrow 2$
- Line 15: $\Theta_{1,2} \leftarrow \{\theta^3, \theta^4\}$
- Line 16: $\mathrm{P}(\theta^3) \leftarrow 1/4$, $\mathrm{P}(\theta^4) \leftarrow 1/4$
- Line 17: $$M_{\Psi}^{X_1}(\Theta_{1,2}) \leftarrow \mathrm{P}(\theta^3)\Big\{\xi_{\theta^3}\left(\psi_{IBR}^{ind}(\Theta_{1,2}; X_1)\right) - \xi_{\theta^3}\left(\psi^{ind}(\theta^3; X_1)\right)\Big\} + \mathrm{P}(\theta^4)\Big\{\xi_{\theta^4}\left(\psi_{IBR}^{ind}(\Theta_{1,2}; X_1)\right) - \xi_{\theta^4}\left(\psi^{ind}(\theta^4; X_1)\right)\Big\}$$
  ✓ we estimated the remaining MOCU when $\theta_1 = 2$ via deleting gene X_1.
- Line 18: $M_{\Psi}^{X_1}(\Theta, 1) \leftarrow \mathrm{P}(\theta_1 = 1)M_{\Psi}^{X_1}(\Theta_{1,1}) + \mathrm{P}(\theta_1 = 2)M_{\Psi}^{X_1}(\Theta_{1,2})$
  ✓ we estimated the expected remaining MOCU when θ_1 is assumed to be known via deleting gene X_1.
- Line 13: $i \leftarrow 2$
- Line 14: $\theta_2 \leftarrow 1$
- Line 15: $\Theta_{2,1} \leftarrow \{\theta^1, \theta^3\}$
- Line 16: $\mathrm{P}(\theta^1) \leftarrow 1/4$, $\mathrm{P}(\theta^3) \leftarrow 1/4$
- Line 17: $$M_{\Psi}^{X_1}(\Theta_{2,1}) \leftarrow \mathrm{P}(\theta^1)\Big\{\xi_{\theta^1}\left(\psi_{IBR}^{ind}(\Theta_{2,1}; X_1)\right) - \xi_{\theta^1}(\psi^{ind}(\theta^1; X_1))\Big\} + \mathrm{P}(\theta^3)\Big\{\xi_{\theta^3}\left(\psi_{IBR}^{ind}(\Theta_{2,1}; X_1)\right) - \xi_{\theta^3}\left(\psi^{ind}(\theta^3; X_1)\right)\Big\}$$
  ✓ we estimated the remaining MOCU when $\theta_2 = 1$ via deleting gene X_1.
- Line 14: $\theta_2 \leftarrow 2$
- Line 15: $\Theta_{2,2} \leftarrow \{\theta^2, \theta^4\}$

- Line 16: $\mathrm{P}(\theta^2) \leftarrow 1/4$, $\mathrm{P}(\theta^4) \leftarrow 1/4$
- Line 17: $$M_{\Psi}^{X_1}(\Theta_{2,2}) \leftarrow \mathrm{P}(\theta^2)\Big\{\xi_{\theta^2}\left(\psi_{IBR}^{ind}(\Theta_{2,2};X_1)\right) - \xi_{\theta^2}\left(\psi^{ind}(\theta^2;X_1)\right)\Big\}$$ $$+\mathrm{P}(\theta^4)\Big\{\xi_{\theta^4}\left(\psi_{IBR}^{ind}(\Theta_{2,2};X_1)\right) - \xi_{\theta^4}\left(\psi^{ind}(\theta^4;X_1)\right)\Big\}$$
  - ✓ we estimated the remaining MOCU when $\theta_2 = 2$ via deleting gene X_1.
- Line 18: $M_{\Psi}^{X_1}(\Theta, 2) \leftarrow \mathrm{P}(\theta_2 = 1)M_{\Psi}^{X_1}(\Theta_{2,1}) + \mathrm{P}(\theta_2 = 2)M_{\Psi}^{X_1}(\Theta_{2,2})$
  - ✓ we estimated the expected remaining MOCU when θ_2 is assumed to be known via deleting gene X_1.
- Line 19: $i^* \leftarrow \underset{i \in 1,2}{\arg\min}\ M_{\Psi}^{X_1}(\Theta, i)$
  - ✓ experiment E_{i^*} which results in the estimation of uncertain regulation θ_{i^*} is the optimal experimented to be conducted first.

B. Box plots for 8-gene networks

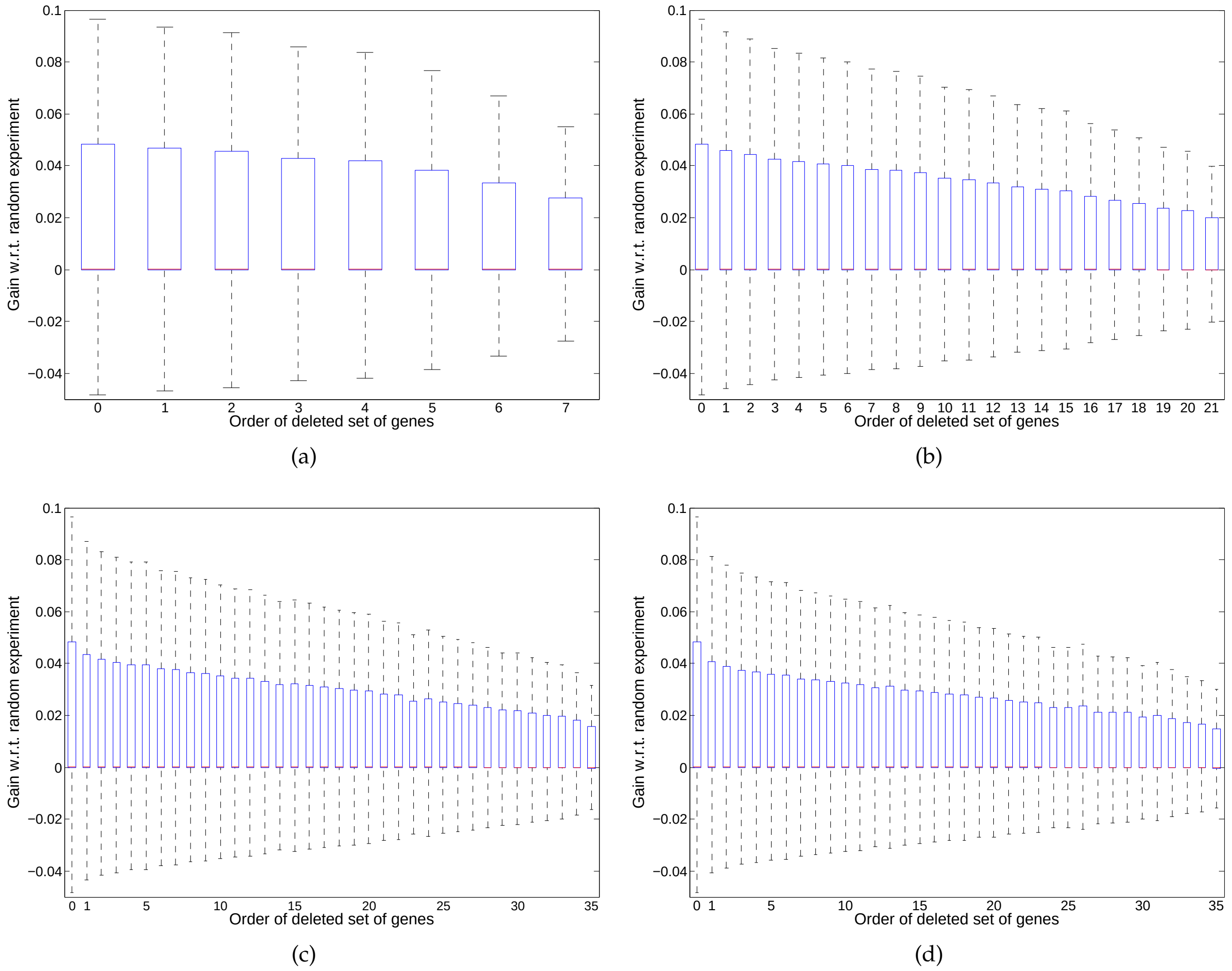

Fig. 3: The box plot of the gain of conducting the chosen experiment by the proposed approximate method with respect to the random experiment when deleting different genes for 8-gene networks with 4 uncertain regulations. (a) Deleting one gene. (b) Deleting two genes. (c) Deleting three genes. (d) Deleting four genes.

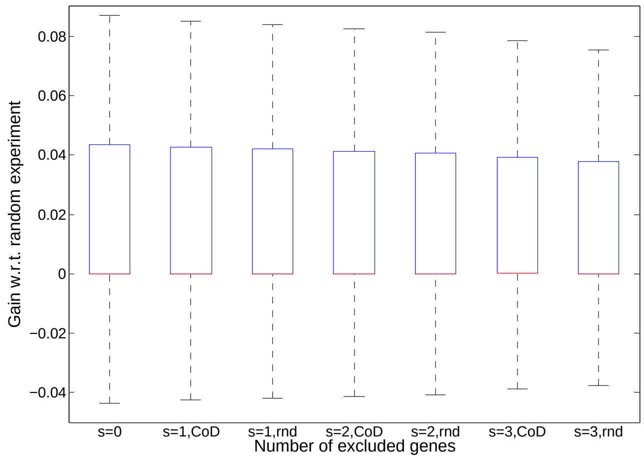

(a)

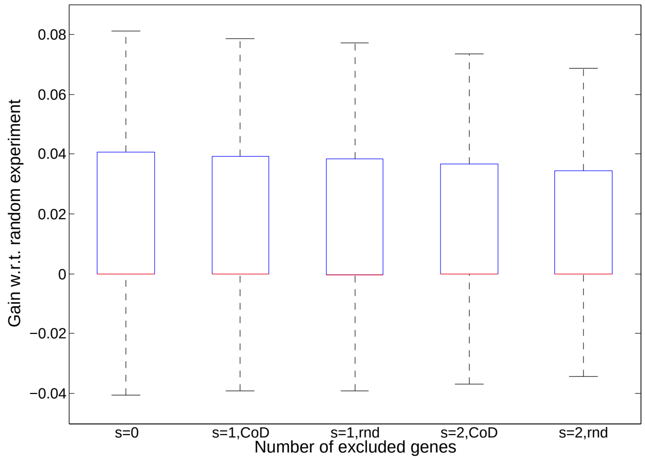

(b)

Fig. 4: The box plot of the gain with respect to the random experiment when s genes are excluded randomly or using the proposed CoD-based procedure. 8-gene networks with 4 uncertain regulations are considered. (a) Deleting three genes. (b) Deleting four genes.
